# *Bacillus* and *Paenibacillus* as plant growth-promoting bacteria in soybean and cannabis

**DOI:** 10.3389/fpls.2025.1529859

**Published:** 2025-06-02

**Authors:** Haleema Tariq, Sowmyalakshmi Subramanian, Anja Geitmann, Donald L. Smith

**Affiliations:** Department of Plant Science, McGill University, QC, Montreal, QC, Canada

**Keywords:** *Bacillus*, cannabis, plant growth promoting bacteria (PGPB), *Paenibacillus*, plant growth, soybean

## Abstract

Many agrochemicals designed to help plants withstand abiotic and biotic stresses can negatively impact the environment. Soil, as an essential natural resource, offers plants organic matter, nutrients, and microbial diversity to thrive in challenging environmental conditions. The soil contains plant growth-promoting bacteria that play an important role in plant/crop productivity, assisting plants through a variety of mechanisms, including nitrogen fixation, phosphate solubilization, phytohormone production, induction of resistance against a wide range of pathogens, and production of microbe-to-plant signals that regulate aspects of plant responses to stress. Plant growth-promoting bacteria such as *Bacillus* and *Paenibacillus* can contribute to sustainable agriculture by enhancing nutrient uptake, acting as biocontrol agents, and producing lytic enzymes with the potential to disrupt or destroy pathogenic organisms in important agricultural and medicinal crops including soybean and cannabis. This review explores the mechanisms of action of plant growth-promoting bacteria, focusing on *Bacillus* and *Paenibacillus* species, and their potential to enhance, optimize plant growth and promote sustainable agriculture.

## 1 Introduction

Major crop plants are challenged by biotic stressors, which include attacks from pathogens such as fungi, bacteria, and oomycetes, and/or abiotic stresses such as radiation, salinity, flooding, drought, extreme temperatures, pH, and heavy metals (Wahab et al., 2023). These stressors cause yield reductions in major crop plants throughout the world. Synthetic agrochemicals like fertilizers and pesticides used to combat the impact of stress are expensive, cause greenhouse gas emissions, leave chemical residues on food crops, and release hazardous chemicals into the environment (Mitra et al., 2021). The need for sustainable food production to sustain a growing human population calls for alternatives to agrochemicals, such as plant growth promoting bacteria (PGPB) that can be used to control biotic and mitigate abiotic stresses (De Andrade et al., 2023). PGPB have the potential to support future sustainable agricultural crop production due to their relatively low impact on native soil microorganisms, livestock, and humans, and minimal impact on soil ecology and biodiversity. Their use in integrated pest management systems has proven to be a sustainable alternative to chemical pesticides, offering a safer, eco-friendly approach to managing plant diseases (Arkhipov et al., 2024).

Plants are associated with beneficial microbial communities located on or inside tissues and are present throughout their developmental cycle. These communities include bacteria, archaea, fungi, and algae, constituting the phytomicrobiome. The portion of the soil close to the roots that is subject to nutritional interference from the roots is named rhizosphere. Plants perform photosynthesis, and depending on the plant species, they invest 10 to 40% of their photosynthetic metabolites in the rhizosphere through rhizodeposition (Vetterlein et al., 2020). Through rhizodeposition, the rhizospheric soil is fertilized and enriched with nutrients, amino acids, and organic energetic molecules such as carbohydrates. Fertilization of the rhizosphere exerts a significant influence and changes the soil microbiota near the roots (Hassan et al., 2019). Plants modulate rhizospheric microorganisms through plant physiological factors that govern plant–microorganisms interactions and by the composition of their exudates. The composition of the microorganisms in the roots is influenced by these root exudates, and the population of the root microbiome takes place in two stages. The first stage is rhizosphere colonization, accomplished by a subgroup of microorganisms from the non-rhizosphere soil and bulk soil. In the second stage, the phyllosphere and endosphere are colonized by a subset of microorganisms from the rhizosphere (Compant et al., 2021; De Andrade et al., 2023). The phytomicrobiome is comprised of the rhizome-microbiome, made up of root-associated microbes, and the phyllo-microbiome, comprised of shoot-associated microbial communities. Depending on their location on the plant surface or in internal tissues, they can also be classified as ecto-microbiome and endo-microbiome, respectively (Kumawat et al., 2023; Vimal et al., 2024). The plant and its phytomicrobiome form the holobiont; it is comprised of numerous beneficial phytomicrobiome members that bolster the plant’s ability to survive in biotic and abiotic stress environments through a variety of mechanisms (Taghinasab and Jabaji, 2020; Baedke et al., 2020; Khan, 2023). *Paenibacillus triticisoli, Rhizobium* sp.*, Pseudomonas* sp.*, Agrobacterium tumefaciens, Azospirillum lipoferum, Azospirillum brasilense, Azoarcus* sp., and *Zoogloea* sp. are few examples of rhizospheric bacteria (Suleiman et al., 2019; Li et al., 2021; Zeng et al., 2022). *Acinetobacter, Enterobacter*, and *Pseudomonas* are the most abundant genera in the roots, stems and leaves (Dong et al., 2019; Firrincieli et al., 2020). Plant growth-promoting bacteria (PGPB) help mitigate the effects of abiotic stresses including salinity, drought, heavy metal stress and acidity which are major hurdles to agricultural production (Jain et al., 2023). Some PGPB act directly by improving the nutritional status of plants via phytonutrients such as fixed nitrogen or solubilized minerals from soil (P, K, Zn, Fe and other essential nutrients) and regulating the levels of plant phytohormone (auxins, cytokinins, gibberellins, abscisic acid and ethylene) (Odoh et al., 2020; Bhat et al., 2023). Other beneficial strains affect plant growth indirectly by suppressing phytopathogens and other deleterious microorganisms through parasitism, competing for nutrients or production of antagonistic compounds (hydrogen cyanide, siderophores, antimicrobial metabolites and antibiotics) (De Ron et al., 2024; El-Saadony et al., 2022). Certain plant growth promoting rhizobacteria produce lytic enzymes such as chitinase, glucanases, and proteases, which induce systemic resistance against foliar and root pathogens. Microbial-derived compounds can also play an important role in the mitigation of abiotic stress effects on plants (Morcillo and Manzanera, 2021), including those associated with climate change. **Table 1** summarizes the mechanisms of action of various PGPB under optimal and stress conditions in various crops. This review aims to examine the mechanisms of action of plant-beneficial bacteria such as *Bacillus* and *Paenibacillus* species, their deployment for plant growth promotion, and their use to optimize plant growth in soybean, cannabis and other agricultural crops.

**Table 1:** Mechanism of action of various PGPB.

PGPR	Mode of action	Crop	Reference
*Bacillus velezensis*	Inhibition of motility traits of *Ralstonia solanacearum*, and damages the pathogen's cell wall through production of fengycin	Tomato	Wang et al., 2023
*Paenibacillus mucilaginosus*	Biofilm formation, solubilization of inorganic and organic phosphate, production of IAA	Tomato	Wang et al., 2023
*Bacillus & Paenibacillus* sp.	Intreases plant growth by aiding the uptake of P, N, K, Fe, and Zn	Maize	Ahmad et al., 2023
*Pseudomonas fluorescens*	Induces systemic resistance against sheath blight	Rice	Renganathan, 2020
*Azoarcus*	Nitrogen fixation	Rice	Fernández-Llamosas et al., 2020
*Bacillus mucilagenosus* & *Bacillus edaphicus*	Potassium solubilization	Peppery, cucumber	Ali et al., 2021
*Pseudomonas putida*	Antibiotic production	Bean	Abo-Elyousr et al., 2021
*Paenibacillus* sp.	Increases biomass, photosynthesis rate of plants, and nitrogen fixation mediated by *nifH* gene	White clover	Li et al., 2022
*Rhizobia*	Hydrogen cyanide production	Soybean	Zoundji et al., 2020
*Rhizobia* sp.	Induces stress resistance in plants	Legumes	Etesami and Adl, 2020
*Pseudomonas aeruginosa*	Produces toxic volatile compounds against *Fusarium oxysporum*	Egg plant	Chandra et al., 2020
*Bacillus endophyticus &* *Pseudomonas aeruginosa*	Modulates the production of IAA, SA, ABA in plants against *Spodoptera litura*	Tomato	Kousar et al., 2020
*Paenibacillus polymyxa*	Solublization of phosphate under varying pH, temperature, and heavy metal stress	Zea mays	Rosli et al., 2020
*Bacillus & Pseudomonas* sp.	Promotes plant growth, increase above ground biomass and yield, and modulate rhizosphere microbiome	Cannabis	Comeau et al., 2021
*Bacillus subtilis BS-2301*	Promotes plant growth and synthesizes IAA during infection with *Sclerotinia sclerotiorum*	Soybean	Ayaz et al., 2024

## 2 Workflow for isolation and identification of plant growth-promoting bacteria

**Figure 1:**
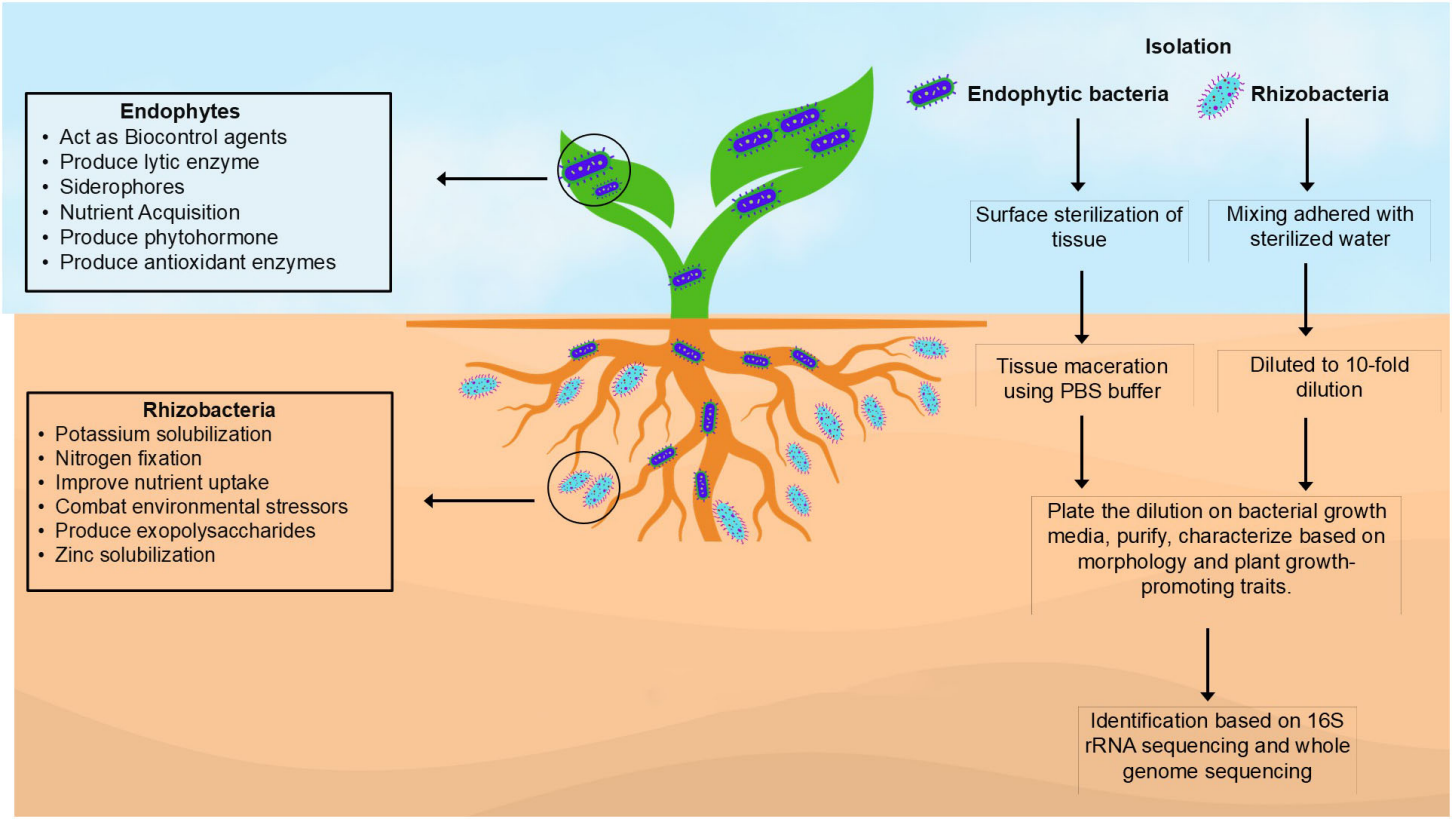
Methods for isolation and identification of PGPB.

Effective screening practices are essential for identifying efficient PGPB. It is therefore imperative to isolate and characterize PGPB to determine their mechanisms that confer them the ability to act as biostimulants, biofertilizers, and bioprotectants. PGPB are primarily identified based on the genomic techniques, which are essential in the deployment of these bacteria in commercial agriculture for bio-protection and yield enhancement. Several methodologies have been used to isolate PGPB. Fan et al. (2016, 2018) employ a comprehensive methodology to isolate rhizobacteria (from soil) by brushing the adhered soil from the roots, dissolving the soil in sterilized water, and diluting 10-fold before plating on different bacterial growth media. Using the sterilization method of Qin et al. (2009), endophytic bacteria can be isolated from surface-sterilized tissues of plants by grinding the tissues in a phosphate buffer solution and centrifuging. The obtained supernatant is diluted 10-fold and plated on agar to recover colonies of endophytes (Fan et al., 2023; Di et al., 2023). **Figure 1** illustrates the step-by-step methodology of isolation of endophytic and rhizobacteria and highlight their important functions. Characterization and identification of PGPR has been done with PCR-based genomic fingerprinting. Williams et al. (1990) introduced the random amplified polymorphic DNA (RAPD) assay, also known as arbitrary primed PCR. The RAPD assay consists of short primers, 9 to 10 bases in length, capable of hybridizing to DNA sequences with sufficient affinity at low annealing temperatures to amplify bacterial genome regions (Boopathi and Boopathi, 2020). Whole genome techniques, such as amplified fragment length polymorphism (AFLP), have been shown to be relatively robust and discriminatory (Louws et al., 1995; Arif et al., 2010). Restriction fragment length polymorphism (RFLP), also known as amplified ribosomal DNA restriction analysis (ARDRA) is another tool applicable to the study of microbial diversity that relies on DNA polymorphism. Rep-PCR (repetitive-sequence-based polymerase chain reaction) is one of the genomic fingerprinting techniques that is used for identification and taxonomy (Versalovic et al., 1991; Rademaker et al., 2004). PCR is used to amplify DNA sequences located between interspersed repeated sequences in prokaryotic genomes. DNA primers designed to complement naturally occurring repetitive sequences are used in Rep-PCR fingerprints, which are found in multiple copies in the genomes of many Gram-negative and Gram-positive bacteria (Lupski and Weeinstock, 1992; Fakruddin et al., 2013; Janczarek and Gałązka, 2024; Matys et al., 2024). These modern genomic techniques can help identify bacteria that can promote plant growth, leading to the deployment of beneficial microbes in sustainable agriculture production.

The culturable bacterial genera considered to be responsible for plant growth promotion include *Acinetobacter, Agrobacterium, Arthobacter, Azotobacter, Azospirillum, Burkholderia, Bradyrhizobium, Rhizobium, Frankia, Serratia, Thiobacillus, Pseudomonas*, and *Bacillus* (Pattnaik et al., 2019). Recent studies have shown that *Bacillus* and *Paenibacillus* sp. have several advantages over other PGPB strains when it comes to formulation of inoculants, long-term maintenance in rhizosphere soil, and potential in sustainable crop production (De Araújo et al., 2011; Orozco-Mosqueda et al., 2021; Saeed et al., 2021). Several species belonging to *Bacillus* and *Paenibacillus* genera are isolated from rhizosphere and plant tissues that can stimulate plant growth directly by synthesizing plant hormones or by increasing mineral nutrient uptake by fixing atmospheric nitrogen, soluble soil phosphorus, and other known mechanisms. In some species, antibiotic metabolites are produced to suppress plant pathogens, whereas other species are known to stimulate plant host defense before pathogen infection. *Paenibacillus* and *Bacillus* sp. colonize the host tissues and biofilm formation improved the bacterium’s ability to act as a biocontrol agent against plant pathogens (Govindasamy et al., 2011; Khan et al., 2020; Li et al., 2022).

Through the green revolution of the 20th century, food production increased significantly; in at least some cases environmental concerns were overcome, and agriculture became more sustainable (Hyder et al., 2024). This led to innovation and the development of a “Fresh” Green Revolution, which has already gone some distance to reducing environmental impacts. One of the main inputs of the more recent bio-revolution involves utilization of PGPB (bio-inoculum, and bioactive compounds of inoculum) and improved crop yield through manipulation of phytomicrobiome structure (Pastor-Bueis et al., 2021; Pandey et al., 2023). As part of this review, the significant potential of the deployment of *Bacillus* and *Paenibacillus* genera is explored, highlighting the various mechanisms that have proven both genera to be effective in promoting plant growth, increasing yields, and improving stress resistance in soybean, cannabis and other crop plants.

## 3 *Bacillus* and *Paenibacillus*

Bacteria that are rod-shaped and can produce endospores under aerobic conditions are known as *Bacillus.* This group has been assigned as a genus because of sporulation properties and it comprises a diverse collection of species (Logan and Halket, 2011; Soares et al., 2023). Taxonomically, *Bacillus* and *Paenibacillus* fall under gram-positive, aerobic, or facultative endospore-forming bacteria but over the past decade advances in 16S rRNA oligonucleotide cataloguing and 16S rRNA sequence analysis revealed that the genus *Bacillus* is phylogenetically very heterogeneous. Consequently, there have been considerable changes in its taxonomy since the first description. The genus *Bacillus* is divided into two groups vernacularly i.e., the *B. subtilis* group and the *B. cereus* group (Claus and Fritz, 1989; Govindasamy et al., 2011). 16S rRNA studies allowed phylogenetic grouping and reclassification. Subsequently, phylogenetic analyses were carried out using other genes and proteins. Patel and Gupta (2020) conducted phylogenetic and comparative genetic sequence analyses defining the members of the *subtilis* and *cereus* clades as well as six additional genera: *Alkalihalobacillus, Cytobacillus, Neobacillus, Mesobacillus, Metabacillus*, and *Peribacillus* and proposed that new species added to the genus *Bacillus* should meet the minimum criteria of the *subtilis* or *cereus* clades and be supported by a phylogenetic tree based on 16S rRNA sequences or by concatenated protein sequences. The “*subtilis* clade”, originally composed of *Bacillus subtilis, B. licheniformis, B. pumilus*, and *B. amyloliquefaciens*, has since been expanded to include several other species (Xu and Kovács, 2024). The “*cereus* clade” comprises the pathogenic species and strains of the genus, namely *B. anthracis*, causing the fatal anthrax disease; *B. cereus*, a foodborne pathogen; and *B. thuringiensis*, an entomopathogen, along with other non-pathogenic species with significant applications in agriculture and industry. There are currently 435 species and 12 subspecies of *Bacillus* in the genus (with verified publications and correct nomenclature) (Parte, 2014), and this genus is a subject to constant modifications (Blanco Crivelli et al., 2024) with advancement and refinement in instrumentation and techniques for in-depth identification.

**Figure 2:**
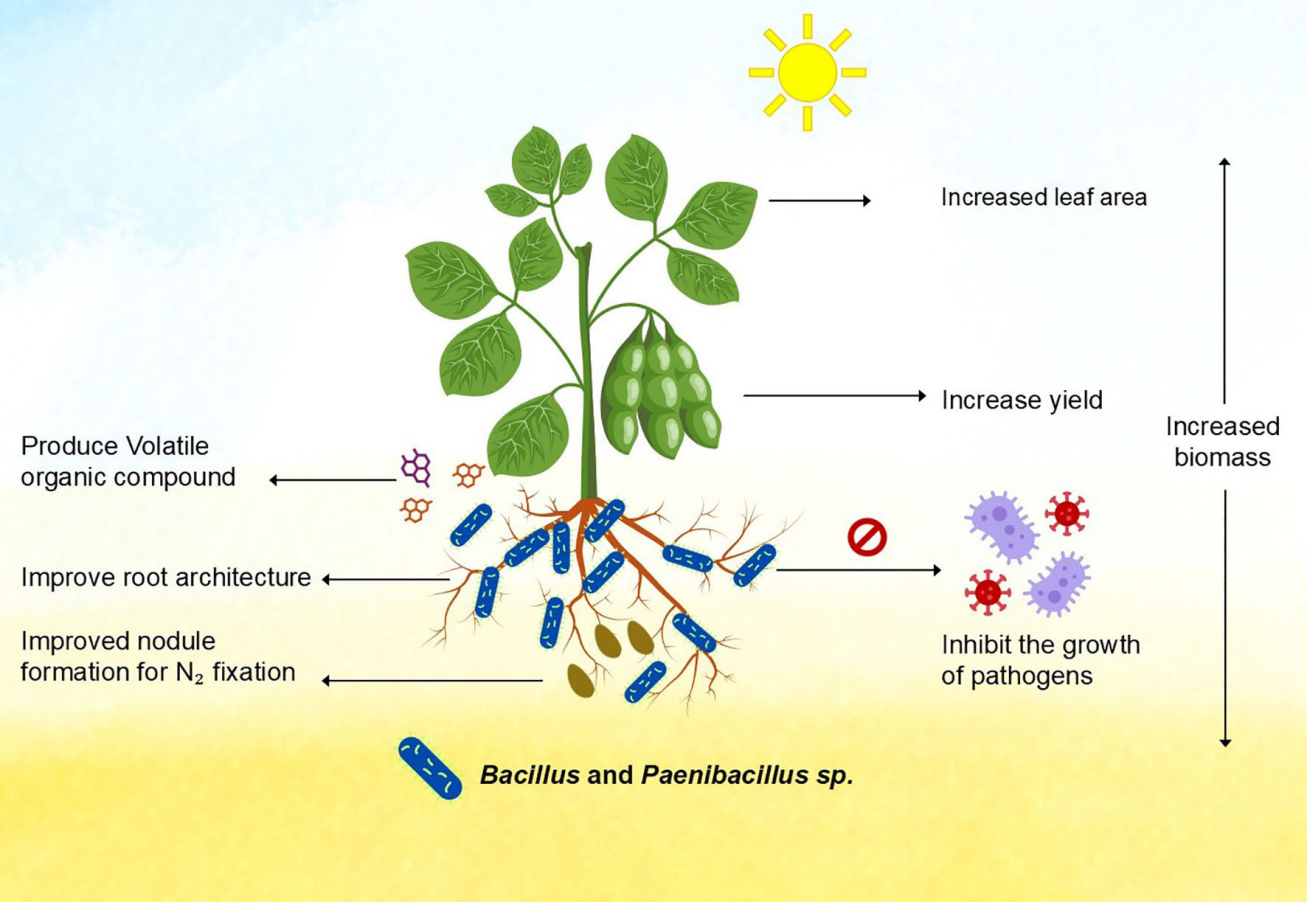
*Bacillus* and *Paenibacillus* promote plant growth.

*Paenibacillus* species were originally included in the genus based on morphological characteristics in common with the type species *B. subtilis*, isolated in 1872 (Zeigler, 2013). However, these characteristics are very ancient and cannot be used to group species into a single genus. Analysis of 188-unit characters suggested that *Bacillus* may be divided into several genera (Priest et al., 1988). In 1991, when 16S rRNA gene sequences were determined to do phylogenetic analyses, it was found that these sequences segregated into at least five distinct clusters, one of which was reassigned to the novel genus *Paenibacillus* in 1993 and includes the type species *Paenibacillus polymyxa* (Ash et al., 1991, 1993). The name *Paenibacillus* is derived from the Latin adverb ‘*paene*’, meaning ‘almost’—almost a *Bacillus*. Several organisms previously classified as separate *Paenibacillus* species were classified as equals shortly after their identification. Among other characteristics, a proposed amendment is described. Species of this genus can be gram-positive, gram-negative, or gram variable while sharing *Bacillus* basal characteristics (Shida et al., 1997; Grady et al., 2016). Meanwhile, novel species of *Paenibacillus* are being discovered and classified; the genus currently contains approximately 200 species (Grady et al., 2016; Lee et al., 2022). **Figure 2** summarizes the various functions of *Bacillus* and *Paenibacillus* genera and their roles in plant growth promotion.

### 3.1 Plant growth promotion perspective of *Bacillus* and *Paenibacillus* in soybean and cannabis

Several agricultural and medicinal crops have been demonstrated to benefit from the use of *Paenbacillus* spp and *Bacillus* spp to promote plant growth and yield, including wheat, maize, soybean, sunflower, common bean, tomato, pepper, potato, cucumber, and cannabis (Aloo et al., 2019; Miljaković et al., 2020; Lyu et al., 2022). Here we focus on using *Bacillus* and *Paenibacillus* species on two important crops: soybean and cannabis. *Bacillus* sp. and *Paenibacillus* sp. stimulate the growth of these crops by increasing nutrient availability, improving soil structure, and inhibiting pathogens (our unpublished data).

#### 3.1.1 Soybean

Soybean offers one of the most important protein sources among legume crops, and plays an important role in the human diet, food, and oil production. Approximately 80% of the soybean sown area in the world is concentrated in three countries: Brazil, the United States, and Argentina (Volkova and Smolyaninova, 2024). Canada currently ranks seventh in the world in terms of soybean production. The soybean planting area is expected to be expanded to 10 million acres (approximately 4 million hectares) by 2027 under an ambitious plan developed by Soy Canada (Soy Canada, 2023). To achieve this target the symbiotic relationship with beneficial microbes like *Bacillus*, and *Paenibacillus* can be leveraged to enhance the ability of soybean to resist abiotic and biotic stresses such as salinity, drought, and heavy metal toxicity and to enhance overall production. The genomic and metagenome analysis of the soybean endosphere reveals that the most dominant group of bacteria in its endosphere is *Streptomyces*, followed by *Chryseobacterium*, *Paenibacillus*, *Bacillus*, and *Mitsuaria.* These species play a role in a variety of biological pathways, including CMP-KDO biosynthesis II (from D-arabinose 5-phosphate), TCA cycle (plant), citrate cycle (TCA cycle), fatty acid biosynthesis, and glyoxylate and dicarboxylate metabolism (Chouhan et al., 2023). *Bacillus* and *Paenibacillus* play important roles in nitrogen fixation, priming of defense mechanisms, up- and down-regulation of various genes and proteins to make soybean resilient to various stress and improve its sustainable production for food security. **Figure 2** summarizes effects of *Bacillus* and *Paenibacillus* sp. and addresses the role of these bacteria in increasing nodule formation, plant growth, biomass production and inhibiting the growth of pathogens in soybean.

##### 3.1.1.1 Role of *Bacillus* and *Paenibacillus* in nitrogen fixation and yield improvement of soybean

Nitrogen fixation is an important ecological process in legumes, including soybean, that is facilitated by a variety of microbes, notably *Bacillus* strains. A rhizobial gene known as nodABC encodes an enzyme responsible for lipo-chitooligosaccharide synthesis, which induces symbiotic responses within the host. As a result of this process, atmospheric nitrogen (N_2_) is converted into physiologically useful ammonium ions (NH^4+^) in the Earth’s nitrogen cycle (Rana et al., 2023). Some *Bacilli* produce nitrogenase, which is responsible for catalyzing nitrogen fixation into ammonia. Plants may directly absorb this ammonia, making it a key source of nitrogen for their growth and development. The ability to fix N_2_ is widely dispersed among bacteria belonging to different phylogenetic groups. The nitrogenase enzyme complex is composed of two proteins: iron (Fe) and molybdenum iron (MoFe), encoded by the *nifH* and *nifDK* genes, respectively. The *nifH* gene sequence has been evolutionarily conserved and is now considered as a marker for N_2_ fixation in *Bacillus* and *Paenibacillus* sp (Mehta et al., 2003; Jain et al., 2021; Li et al., 2021). Vitorino et al. (2024) reported that *Bacillus velezensis* enhances root growth and increases nodules and flowers, resulting in a positive impact on grain yield, phosphate content, and weight in soybean. *B. aerophilus* and *B. subtilis* improved soybean production, specifically seed dry weight, N uptake, and the number of soybean root nodules (Nuraini et al., 2024). *Bacillus* strains form symbiotic relationships with specific plants, known as nitrogen-fixing nodules in legumes, where they provide the host plant with a direct source of ammonia contributing to the development of plants. This mutualistic relationship also facilitates the development of sustainable agricultural practices through the reduction of nitrogenous fertilizer use (Fahde et al., 2023; Iturralde et al., 2019). *Bacillus thuringiensis* produces a peptide known as thuricin-17, which increases soybean tolerance to drought when used with *Bradyrhizobium japonicum*, modifies below-ground structures, increases root and nodule biomass, slightly increases leaf area and photosynthetic rate (Prudent et al., 2015). The *Bacillus amyloliqueficaciens* EB2003A releases bioactive compounds in its growth media that enhance corn and soybean radicle length and percentage germination under optimal and NaCl-stressed growth conditions (Naamala et al., 2022).

The *Paenibacillus nif* operon consists of a cluster of genes that encode *nif*, measuring 11 kb in size and demonstrating that it is capable of fixing nitrogen (Dasgupta et al., 2021). Nitrogen fixers such as *P. azotofixans, P. macerans, P. polymyxa, P. graminis*, and *P. odorifer* contain the *nifH* gene (Prabhukarthikeyan et al., 2022). Ribeiro et al. (2024) reported insights related to alternative N_2_-fixation by Fe-only nitrogenase in *P. sonchi* and also observed endospore formation during N_2_-fixation in *P. durus*, coinciding with the highest levels of *nifH* transcription. Plant growth stimulation and an increase in shoot and root dry weight were observed after inoculation with *P. polymyxa*-*gfp* strain alone and in combination with *B. japonicum*. *In vivo* simultaneous visualization using Confocal Laser Scanning Microscopy (CLSM) demonstrated that *P. polymyxa* invades soybean roots and root nodules and improves plant growth when inoculated individually or in combination with *B. japonicum* (Annapurna et al., 2013). The *Bacillus* and *Paenibacillus* sp. provide a key mechanism of nitrogen fixation for the resilience of soybean in the face of abiotic and biotic stresses.

##### 3.1.1.2 *Bacillus* and *Paenibacillus* sp. influence soybean plant metabolomics and proteomics profiles

**Figure 3:**
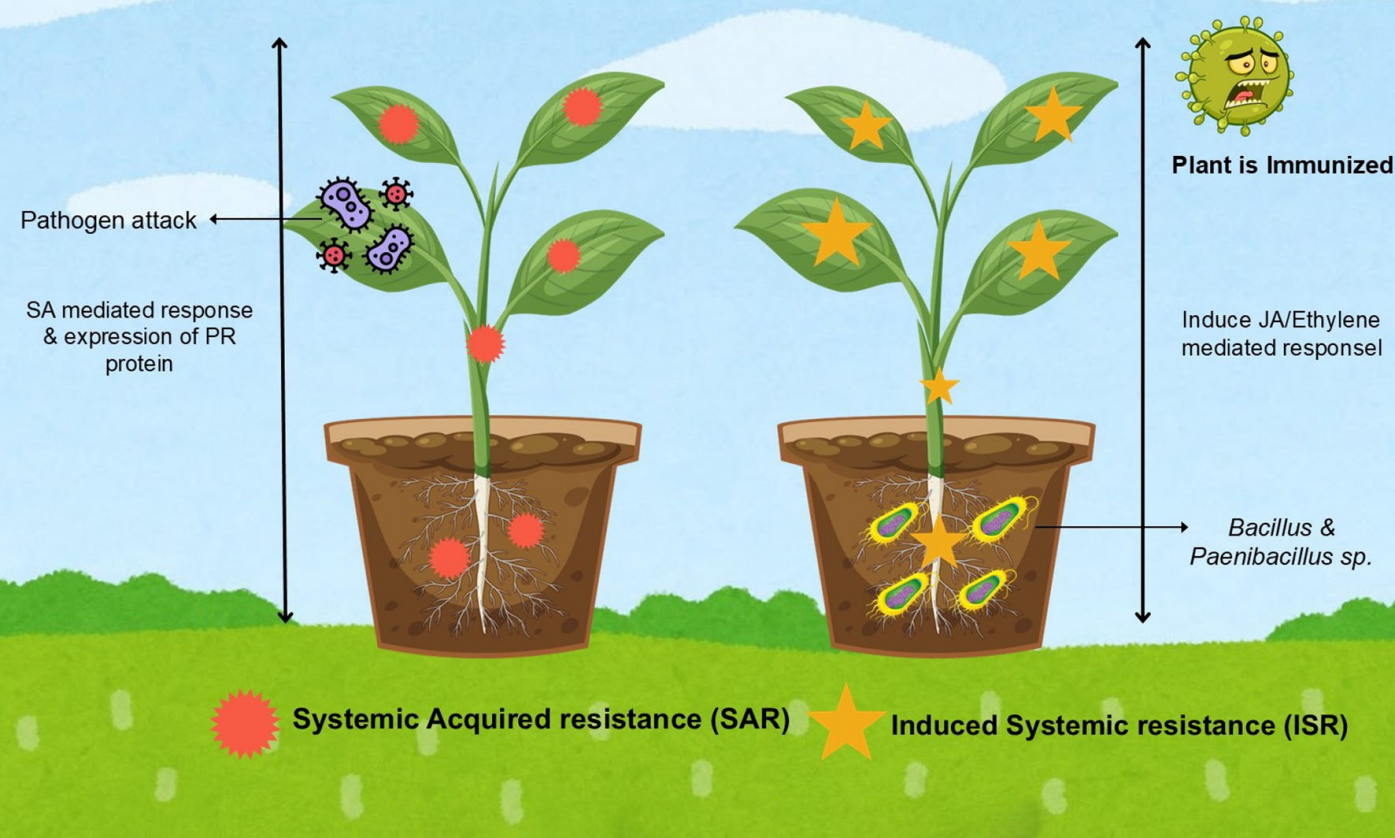
*Bacillus* and *Paenibacillus* as elicitors of ISR.

Signal compounds produced by phytomicrobiome members can enhance plant growth through various mechanisms, including activation of antioxidant metabolism pathways, protein activities, induction of plant defense systems, increased photosynthesis rate, production of phytohormones and modification of plant root systems (Subramanian and Smith, 2015; Lyu et al., 2021). The plant activates the first-layer defense response, called pathogen-associated molecular patterns (PAMP)-triggered immunity (PTI) and initiates local responses where the pathogen attacks and then induces responses in the more distant uninfected plant parts; this induced systemic resistance (ISR) in plants can also be activated by non-pathogenic plant growth promoting bacteria that help in suppressing disease. It is activated through jasmonic acid/ethylene or salicylic acid (SA) signaling (Zhang et al., 2024) (**Figure 3**). In the host plant, ISR modulates many biochemical and cytological processes including the deposition of lignin in the cell wall, the production of phytoalexins, and the synthesis of other antimicrobial substances such as peroxidases and glucanases (Mahapatra et al., 2022). The *Bacillus* and *Paenibacillus* sp. play important roles in modulating soybean defense response. For instance, *Bacillus* and *Paenibacillus* are effective biocontrol agents and produce antimicrobial compounds such as cyclic lipopeptides that can bind membrane lipids, cause structural permeability, and damage fungal cell membranes (Yang et al., 2024; Gómez-De la Cruz et al., 2023).

*Bacillus subtilis* inhibits growth of *Sclerotinia sclerotiorum*, the causative agent of stem white mold disease in soybean. It causes severe oxidative stress to fungal hyphae and inhibits melanin synthesis in the sclerotia (Ayaz et al., 2024). *Bacillus velezensis* inhibits the growth of soybean root rot pathogens and reduces germination, conidia production, and mycelial growth of *F. oxysporum* resulting in hyphal malformations and effectively controlling soybean root rot (Sun et al., 2023). *Bacillus* and *Paenibacillus* species produce indole acetic acid(IAA) as secondary metabolites that aid plant growth. *B. altitudinis* TM22 produces IAA, enhances freshshoot weight, shoot length, fresh root weight, dry shoot weight, and dry root weight and upregulates the expression of growth-related genes such as those related to production of expansin (*EXP-1* and *EXP-2*), cytokinin (*CKX*), auxin (*IAA-1* and *IAA-6*), and gibberellin (*GA20OX-1* and *GA20OX-2*) (Moosa et al., 2024). *Bacillus velezensis* strain (BVPS01) was found to be more efficient at solubilizing phosphates by producing the phosphatase enzyme, as indicated by the expression of the *phoC* and *phoD* genes in soybean. This bacterium has been recognized as a model plant growth-promoting bacterium for field-grown soybean owing to its excellent performance in increasing the growth of soybean and grain yield (Vitorino et al., 2024). As a result of using the signal compounds of *Brabyrhizobium japonicum* and *B. thuringiensis* in combination with optimal and NaCl-stressed seeds of soybean, Subramanian et al. (2016) identified a wide variety of proteins that are known, predicted, hypothetical, and unknown. Under both optimal and salt-stressed conditions, carbon, nitrogen, and energy metabolism pathways were affected by signals. Proteins such as phosphoenolpyruvate carboxylase, rubisco oxygenase large subunit, pyruvate kinase, and isocitrate lyase were enhanced by the signals, along with antioxidant glutathione-S transferase and other stress-related proteins that enhance tolerance or adaptation to salt stress. *Bacillus simplex* alters the soybean root metabolic profile due to the presence of soybean cyst nematodes, resulting in metabolic differences that explain the nematode resistance and *B*. *simplex*-treated roots contained lower levels of glucose, fructose, sucrose, and trehalose, which reduces nematodes’ food sources. Moreover, treatment with *B. simplex* results in higher levels of melibiose, gluconic acid, lactic acid, phytosphingosine, and noradrenaline in soybean roots, which contributes to nematocidal activity. Oxoproline, maltose, and galactose levels are reduced following *B. simplex* treatment, thereby improving disease resistance (Kang et al., 2020). As demonstrated using scanning electron microscopy analysis, *Bacillus aryabhattai* colonizes soybean roots and its presence enhances 18 different amino acids in soybean plants. Matrix-assisted laser desorption ionization–time of flight (MALDI-TOF) mass spectrometry (MS) identified several proteins including ß conglycinin and glycinin that were traced back to their respective genes, and a significant increase in butanoic acid was observed in bacterial culture filtrates. This significant increase in butanoic acid significantly influences plant growth via chlorophyll maintenance (Mun et al., 2024).

The *P. polymyxa* CR1 is capable of priming drought tolerance in *Arabidopsis* and soybean. In addition, it induces the expression of *RD29A* and *RD29B* (memory genes), thus enhancing the plant’s ability to withstand drought without reducing yield potential (Liu et al., 2020). *P. mucilaginosus* and *Bradyrhizobium japonicum* significantly alter soybean rhizobacteria compositions, increase soil-available phosphorus. The phosphatase activity significantly increases soybean biomass, nitrogen and phosphorus content in the rhizosphere (Xing et al., 2022). The influence of *Paenibacillus* on soybean plant metabolomics and proteomics profiles has not been extensively reported; and, there remains a need to investigate the actual mechanism by which these PGPB interact with soybean plants, as well as the effects of these PGPB on soybean plant transcriptomics, proteomics and metablomics.

#### 3.1.2 Cannabis

The cultivation of cannabis has been carried out for both medicinal and industrial purposes for nearly a century, but its illegal status has resulted in limited availability of systematic studies or scientifically underpinned cultivation practices. Recent years have seen an enhanced level of research on this species allowing for knowledge databases to become more robust. Among all the cannabinoids, 9-THC (Δ9-tetrahydrocannabinol) and CBD (cannabidiol) are the major components of cannabis with pharmacological relevance. Clinically, cannabis-derived cannabinoids have been developed and applied as treatment for chronic pain, epilepsy, multiple sclerosis, and cancer, as well as appetite stimulants and antiemetic agents in HIV/AIDS and cancer patients (Bruni et al., 2018; Cristino et al., 2020; Dhiman et al., 2024). Increasing the overall yield of these components is a necessity for the pharmaceutical industry. Abiotic and some biotic stress challenges are reported to adversely affect cannabis productivity and secondary metabolite production. The presence of PGPB, such as *Paenibacillus* and *Bacillus*, plays a significant role in improving cannabis resilience by enhancing root architecture, promoting nutrient acquisition, and reducing stress impacts (Comeau et al., 2021; author unpublished data).

##### 3.1.2.1 Bacillus and Paenibacillus control pathogens of cannabis

*Bacillus* types are effective biocontrol agents and produce antimicrobial compounds such as cyclic lipopeptides that can bind membrane lipids, cause structural permeability, and damage fungal cell membranes (Yang et al., 2024). Phytomicrobiome members can inhibit the growth of pathogenic microorganisms by producing signals that are bacteriocins (small proteins/peptides such as thuricin 17), to remove competitors from niche space and promote plant growth, increasing the niche space, for this signal producing bacterium (Nazari and Smith, 2020). Bacteriocins are ribosomally synthesized peptides that inhibit the growth of microbial organisms by binding with membrane phospholipids, forming non-specific ion channels and forming pores causing cell death, and by other -cidal or -static mechanisms (Vasilchenko and Valyshev, 2019).

*Bacillus* sp. increases the seed germination rate of cannabis cultivars and exhibits antagonistic activities towards mycelial growth of the cannabis pathogen *F. oxysporum* (Corredor-Perilla et al., 2023). Balthazar et al. (2022) reported that *B. velezensis*, *B. subtilis* and *P. protegens* are biocontrol agents against *B. cinerea* of cannabis and exert beneficial effects through antibiosis in the phyllosphere. Afzal et al. (2015) reported that the endophytic strains of *Paenibacillus* sp. and *Pantoea vagans* successfully antagonize the pathogen *Fusarium oxysporum* in dual confrontation assays and produce fungal cell wall degrading enzymes. Cannabis accessions possess seed-inherited *P. mobilis* with the capacity to solubilize mineral phosphate and *P. polymyxa* that is antagonistic to hemp *Alternaria, Aspergillus, Fusarium, Penicillium* species affecting hemp (Dumigan and Deyholos, 2022). Clearly, the use of *Bacillus* and *Paenbacillus* sp. can help cannabis plants overcome stress challenges, allowing them to be more productive and produce higher secondary metabolite concentrations to mitigate the effects of stressful environments. Due to legal restrictions with regards to the use and research on cannabis, research data on the biocontrol mechanisms of *Bacillus* and *Paenibacillus* species against cannabis pathogens are limited.

##### 3.1.2.2 *Bacillus* and *Paenibacillus* sp. influence cannabis plant metabolomics and proteomics profiles

There are limited studies on the use of PGPB and their effects on cannabis growth and yield. Some bacterial consortia are reported to favor plant growth development and the accumulation of secondary metabolites (i.e., CBD and THC). Jalali et al. (2019) evaluated the effect of SA and γ-aminobutyric acid (GABA) on THCAS (THC-synthase), CBDAS (CBD synthase), OLS (3,5,7-trioxododecanoyl-CoA synthase) and *PT* genes which are responsible for production of the main cannabinoids and found that SA and GABA can control the signaling cascades of genes in the cannabinoid pathway by changing their expression patterns at critical concentration. These two compounds can be considered as effective elicitors for commercial cannabinoid production. Mansouri et al. (2011) analyzed the effect of exogenous gibberellic acid (GA3) on plastidic, and cytosolic terpenoids as well as two key enzymes involved in terpenoid biosynthesis, 1-deoxy-D-xylulose-5-phosphate synthase (DXS) and 3-hydroxy-3-methylglutaryl coenzyme A reductase (HMGR). Leaves of GA3-treated plants contained increased levels of THC and CBD in comparison to control plants. It is hypothesized that exogenous PGPB can induce and will improve secondary metabolite synthesis and recovery. *Bacillus* sp. improved the cannabis yield and quality and increased the levels of cannabinoids and terpenes in *Cannabis sativa* (Lyu et al., 2023). Lyu et al. (2022) evaluated the effect of *Bacillus* sp. along with other PGPB and found that *Bacillus* sp. and *Mucilaginibacter* sp. increase flower number and axillary bud outgrowth rate. In the presence of PGPB, such as *Pseudomonas* sp. and *Bacillus* sp., trichome density was enhanced and major cannabinoid production was influenced, leading to an opportunity to reduce the use of synthetic fertilizers without compromising yield (Tanney et al., 2023).

Several studies have surveyed the diversity of bacterial and fungal endophytes in medical/recreational cannabis and hemp and have found that colonization depends on the cannabis chemovar, the plant tissue sampled, and the timing of sample collection relative to the plant growth stage. The most common bacterial genera associated with medical/recreational cannabis and hemp plants were *Pseudomonas, Staphylococcus, Bacillus, Acinetobacter, Chryseobacterium, Enterobacter*, and *Microbacterium*. *Erwinia*, and *Cedecia. Chryseobacterium* and *Enterobacter* were typically detected at lower frequencies (Winston et al., 2014; Scott et al., 2018; Backer et al., 2019, 2020). However, detailed knowledge is lacking because of cannabis being illegal for centuries which presented a major barrier to research. For instance, no study has investigated the interaction of *Paenibacillus* sp. and cannabis plants and how the *Paenibacillus* sp. affects the metabolic and protein profile of cannabis plants.

## 4 Plant growth promotion perspective of *Bacillus* and *Paenibacillus* species in other crops

### 4.1 *Bacillus* and *Paenibacillus* as nitrogen fixers

Species of *Bacillus* are involved in promoting plant growth and development and reducing the effects of environmental stress factors, such as prolonged drought, salinity, high temperatures, metal pollution, toxicity, and flooding (Etesami et al., 2023). Kour and Yadav (2022) reported that *B. cereus* strain BEB1, *B. cereus* strain BEB2, *B. tropicus* strain BEB3 and *B. thuringiensis* strain BEB4 showed significant growth on nitrogen-free malate media by atmospheric nitrogen fixation. Several *Bacillus* species including *B. megaterium*, *B. coagulans, B. pumilus*, *B. circulans*, *B. licheniformis*, *B. subtilis*, *B. brevis*, and *B. firmus* were found to be N_2_-fixing bacteria, based on their nitrogenase activity (Kaymak et al., 2023).

The *Paenibacillus* nif operon consists of a cluster of genes that encode *nif*, measuring 11 kb in size and demonstrating that it is capable of fixing nitrogen (Dasgupta et al., 2021). Nitrogen fixers such as *Paenibacillus azotofixans, P. macerans, P. polymyxa, P. graminis*, and *P. odorifer* contain the *nifH* gene (Prabhukarthikeyan et al., 2022). Ribeiro et al. (2024) reported insights related to alternative N_2_-fixation by Fe-only nitrogenase in *P. sonchi* and also observed endospore formation during N_2_-fixation in *Paenibacillus durus*, coinciding with the highest levels of *nifH* transcription. NH_4_^+^ and nitrate (NO_3_^-^) uptake from soil was enhanced by inoculation with *P. beijingensis* BJ-18 especially in low soil nitrogen conditions. Enhanced gene expression and enzyme activities involved in N uptake and assimilation in plants were also reported (Li et al., 2019). The presence of *P. triticisoli* in the rhizosphere significantly increased total soil N, available P, nitrogenase activity, wheat yield, and the number of *nif* genes in the rhizosphere (Li et al., 2021).

### 4.2 *Bacillus* and *Paenibacillus* as biocontrol

*Bacillus* and *Paenibacillus* species produce broad-spectrum peptide antibiotics active against numerous pathogens and nematodes. *Bacillus velezensis* strains inhibit the growth of *B. cinerea* by producing antifungal compounds such as iturin A2, surfactin-C13 and -C15, oxydifficidin, bacillibactin, L-dihydroanticapsin in grape berries (Nifakos et al., 2021). Volatile organic compounds (VOCs) produced by one organism can travel long distances and act as biocontrol agents (Schulz-Bohm et al., 2017). One such example is that of *B. velezensis* which produces pyrazine, benzothiazole, and phenol against *B. cinerea* (Wang et al., 2022). Using scanning electron microscopy, Salvatierra-Martinez et al. (2018) reported that foliar spray of *B. amyloliquefaciens* can alter mycelial growth of *B. cinerea* in tomato leaves, while it promotes plant growth when applied through root drench by producing IAA and 2,3-butanediol. The strain *B. amyloliquefaciens* BBC047 has the ability to produce complex biofilms, improved plant resistance to pathogens and maintains an elevated population density over time on tomato leaves (Legein et al., 2020). *Bacillus* sp. are effective biocontrol agents against *Ralstonia solanacearum*, capable of significantly reducing the severity of ginger bacterial wilt and enhancing plant growth in ginger (Cui et al., 2024). The *B. velezensis* Y6 is believed to suppress potato scab caused by *Streptomyces scabies* by secreting lipopeptides (surfactin and iturin) and stimulating potato root growth by increasing the expression of genes that are involved in cell wall organization and biogenesis (Tao et al., 2023).

*Paenibacillus polymyxa* produces amylase, pectinase, and cellulase enzymes and inhibit the growth of the *Xanthomonas translucens* and *Fusarium graminearum* and increase grain weight, chlorophyll content, and carotenoid levels in wheat (Taheri et al., 2022). *P. chitinolyticus*, produces high levels of chitinase, and suppresses *Plasmodiphora brassicae* by targeting chitin in a critical stage of its life cycle, decreases the disease index significantly within *Brassica* species, and increases shoot dry weight (Khodashenas Rudsari et al., 2024). *P. polymyxa* PJH16 protects plants from *Fusarium* cucumber disease by forming biofilms in plant roots and resisting pathogens; this species produces a variety of hydrolases and antimicrobial lipopeptides that act on the fungal cell wall and directly inhibit the growth of pathogenic fungi. It also secretes IAA and siderophores to promote plant growth and resist pathogens (Yang et al., 2024). *P. polymyxa* produces paenibacillin which is a post-translationally modified lantibiotic, type B globular lantipeptide, that exhibits broad-spectrum antimicrobial activity against gram-positive bacteria by pore formation in the cytoplasmic membrane (Olishevska et al., 2019).

### 4.3 *Bacillus* and *Paenibacillus* as elicitors of induced systemic resistance

When a pathogen crosses the constitutive plant defensive barrier, the plant must defend itself by activating specific defenses, recognizing the molecular components of pathogen responses, and recognizing the molecular components of PAMPs through pattern recognition receptors (PRRs) (Rampitsch and Bykova, 2012; Tanaka and Heil, 2021). Several lines of evidence suggest that *Bacillus* species induce systemic resistance in plants in response to various biotic stresses, such as fungi, bacteria, viruses, and nematodes. *B. cereus* can trigger the JA/ET- signaling pathway and induce ISR in *Arabidopsis* plants, to act against *B. cinerea*, by reducing necrosis diameter and inhibiting leaf fungal growth (Nie et al., 2017). **Figure 3** summarizes the role of pathogens in inducing SAR and the role of rhizobacteria in ISR. Root inoculation of *B. amyloliquefaciens* increases the expression of *PR1* and β-1,3-glucanase genes, through the SA-dependent pathway in the leaves of strawberries (Kamle et al., 2020). *B. velezensis* reduces disease severity by 50% in tomato leaves through the JA/ET pathway and reduces oxidative damage and callose formation (Toral et al., 2020). A significant increase in the expression of chitinase (PR-3 and PR-4), lipid transfer proteins (PR-14), peroxidase (PR-9), and lipoxygenase (LOX) was observed in vegetable crop plants after treating with *B. subtilis* (Abbas et al., 2019; Kumar et al., 2024). *B. velezensis* types are potential biocontrol agents against *B. cinerea*, releasing VOCs and increasing transcription of three *PR* genes with the ability to activate the SA-mediated defense signaling pathway (Jiang et al., 2018). In maize, *P. polymyxa* strain SF05 induces systemic resistance to maize blight by priming defense genes, producing VOCs, creating biofilms, and upregulation of ZmPR1a in the stem (Chen et al., 2022). *P. polymyxa* J2–4 exhibits excellent biocontrol efficacy against *M. incognita* in cucumber plants and active host defenses; it induces JA and SA signaling responsive gene expression and inhibits nematode development in local and systemic roots (Shi et al., 2024).

### 4.4 *Bacillus* and *Paenibacillus* produce indole acetic acid

Auxins play a role in plant gene expression, development, cell division, cell wall modification, cell elongation, fruit development, senescence, and lateral root formation. The first class of auxins identified and most abundant in nature is IAA. Plants can produce their phytohormones, but they can also use foreign sources provided by other organisms (Bertoni, 2011; Grady et al., 2016; Moreno-Serrano et al., 2024). Tran et al. (2024) reported the evaluation of a novel species of *Paenibacillus* and revealed that a strain produced phytohormones (IAA, GA3, and zeatin), biofilms, and siderophores highlighting the potential of these strains as plant growth‐promoting agents for sustainable crop production. *P. polymyxa* accelerate maize, potato, cucumber, *Arabidopsis*, and tomato growth, utilize atmospheric nitrogen and insoluble phosphorus, produce IAA and degrade and use lignocellulose components (Weselowski et al., 2016). The *P. polymyxa* SK1 strain isolated from *Lilium lancifolium* produces IAA using a tryptophan-dependent pathway and was shown to promote the growth of two *Lilium* varieties (Khan et al., 2020). Sun et al. (2022) reported that *P. polymyxa* can enhance plant growth by directly secreting IAA. The authors identified the native IPyA pathway of IAA synthesis in the strain and evaluated the ability to express IAA synthetic genes using the novel and very effective promoter P04420.

### 4.5 *Bacillus* and *Paenibacillus* change proteomic and metabolomic responses

PGPB including *Bacillus* and *Paenibacillus* have unique methods to deal with environmental stressors and safeguard the host plant by affecting plant proteomics and metabolomics profiles. For instance, biostimulants such as lipo-chito-oligosaccharide (LCO - derived from *Bradyrhizobium japonicum*) and thuricin-17 (Th17 - derived from *Bacillus thuringiensis*) are found to alleviate drought stress by modulating drought-specific proteomics and metabolomic responses. Untargeted proteomics analysis revealed changes in the levels of drought-specific ribosomal proteins, glutathione S-transferase, proteins of late embryogenesis, vegetative storage proteins 1 and 2, thaumatin-like proteins, and proteins involved in chloroplast and carbon metabolism, all of which contribute to mitigating drought stress in *Arabidopsis thaliana*. Targeted metabolomic analysis for phytohormones revealed that LCO-treated rosettes showed decreases in total IAA, cytokinins, gibberellins, and jasmonic acid, and increased levels of ABA and SA whereas Th17-treated rosettes showed an increase in IAA and SA (Subramanian et al., 2023). *B. pumilus* and silicon (Si) increased the tolerance of *Glycyrrhiza uralensis* to drought stress by maintaining the homeostasis of reactive oxygen species (ROS), and results demonstrated that *B. pumilus* and Si enhanced the antioxidant defense system, accelerated the AsA–GSH (ascorbate (AsA)–glutathione) cycle, stimulated carotenoid metabolism, and eliminated excess ROS caused by drought. Daidzein, medicarpin, glycitin, and astragalin downstream metabolites are observed to respond differently to drought stress based on metabolomic analysis. It has been shown that 3-O-methylquercetin, a derivative of quercetin, increases in *B. pumilus* and plants treated with it, suggesting these flavonoids may play a key role in alleviating drought-induced oxidative stress (Ma et al., 2022). There are 41 proteins identified as differentially expressed in roots and shoots of Arabidopsis as a result of interaction with *P. polymyxa.* It was found that *P. polymyxa* improved plant growth by altering proteins related to defense/stress, antioxidant (GST, APX1, GPX6, PER43, CML42, and BAS1), photosynthesis, and plant hormones (auxin signaling-IAA9, HLS1, and ACT7) and tryptophan/camalexin (ASB1, GSTF6, and CYP71B15) biosynthesis-related proteins and metabolomic analyses showed that treated plants had increased levels of tryptophan, indole-3-acetonitrile (IAN), IAA, and camalexin (Kwon et al., 2016).

## 5 Conclusions and future prospectives

Research into the functioning of *Bacillus* and *Paenibacillus* is not only critical for advancing scientific understanding of microbial functions but it is also indispensable for real-world agricultural practices, where these bacteria can be leveraged to significantly enhance plant health and crop productivity. The *Bacillus* and *Paenibacillus* species have the potential to play a crucial role in sustainable agriculture, particularly in cases where climate change, disease, and biotic stressors are affecting our ability to feed an increasingly populous world. In addition to contributing to addressing future food security challenges, *Bacillus* and *Paenibacillus* also allow reducing the environmental impact of agricultural practices and increasing the resilience of food crop productivity against climate change. With this knowledge, it is possible to develop biotechnological products and methods for resolving plant diseases biologically, using microbes that benefit plants as bio-control, stimulate plant growth as biofertilizers, and assist with phytoremediation. To be able to implement *Bacillus* and *Paenibacillus* species practically on a wide scale, a comprehensive understanding of their mechanisms of action is imperative. Recent advances in microbial genetics have led researchers to explore ways to improve the efficiency of these bacteria as biocontrol agents, biofertilizers, and growth promoters. These species offer an opportunity to increase soybean yield and produce secondary metabolites in medicinal plants such as cannabis. PGPR strains must be evaluated concerning one another as well as with chemicals and organic supplements. The development of bioformulations and bioinoculants that can be applied to different soil types and/or foliar applications should also be a focus of future research. The mechanism of interaction between plants, *Paenibacillus*, *Bacillus* species and pathogens need to be explored using omics and imaging technology like metabolomics, proteomics and metagenomics to find out the actual mechanism of action of these bacteria in different crops including soybean and cannabis and their antagonist effect with different pathogens to implement them at a large scale.

## References

[B1] Abbas A. Khan S. U. Khan W. U. Saleh T. A. Khan M. H. U. Ullah S.. (2019). Antagonist effects of strains of *Bacillus* spp. against *Rhizoctonia solani* for their protection against several plant diseases: Alternatives to chemical pesticides. Comptes Rendus. Biologies 342, 124–135. doi: 10.1016/j.crvi.2019.05.002 31402177

[B2] Abo-Elyousr K. A. M. Abdel-Rahim I. R. Almasoudi N. M. Alghamdi S. A. (2021). Native Endophytic *Pseudomonas putida* as a biocontrol agent against common bean rust caused by *Uromyces appendiculatus* . J. Fungi 2021 7, 745. doi: 10.3390/jof7090745 PMC8467904 34575783

[B3] Afzal I. M. R. A. N. Shinwari Z. K. Iqrar I. (2015). Selective isolation and characterization of agriculturally beneficial endophytic bacteria from wild hemp using canola. Pak. J. Bot. 47, 1999–2008. doi: 10.1016/j.micres.2019.02.001

[B4] Ahmad M. Hussain A. Dar A. Luqman M. Ditta A. Iqbal Z.. (2023). Combating iron and zinc malnutrition through mineral biofortification in maize through plant growth promoting *Bacillus* and *Paenibacillus* species. Front. Plant Sci. 13, 1094551. doi: 10.3389/fpls.2022.1094551 36816488 PMC9929565

[B5] Ali A. M. Awad M. Y. Hegab S. A. Gawad A. M. A. E. Eissa M. A. (2021). Effect of potassium solubilizing bacteria (*Bacillus cereus*) on growth and yield of potato. J. Plant Nutr. 44, 411–420. doi: 10.1080/01904167.2020.1822399

[B6] Aloo B. N. Makumba B. A. Mbega E. R. (2019). The potential of *Bacilli* rhizobacteria for sustainable crop production and environmental sustainability. Microbiological Res. 219, 26–39. doi: 10.1016/j.micres.2018.10.011 30642464

[B7] Annapurna K. Ramadoss D. Bose P. VithalKumar L. (2013). *In situ* localization of *Paenibacillus polymyxa* HKA-15 in roots and root nodules of soybean (*Glycine max.* L.). Plant Soil 373, 641–648. doi: 10.1007/s11104-013-1825-7

[B8] Arif I. A. Bakir M. A. Khan H. A. Al Farhan A. H. Al Homaidan A. A. Bahkali A. H.. (2010). A brief review of molecular techniques to assess plant diversity. Int. J. Mol. Sci. 11, 2079–2096. doi: 10.3390/ijms11052079 20559503 PMC2885095

[B9] Arkhipov A. Shao Z. Muirhead S. R. Harry M. S. Batool M. Mirzaee H.. (2024). Microbe-friendly plants enable beneficial interactions with soil rhizosphere bacteria by lowering their defense responses. Plants 13, 3065. doi: 10.3390/plants13213065 39519980 PMC11548416

[B10] Ash C. Farrow J. Wallbanks S. Collins M. (1991). Phylogenetic heterogeneity of the genus *Bacillus* revealed by comparative analysis of small-subunit ribosomal RNA sequences. Lett. Appl. Microbiol. 13, 202–206. doi: 10.1111/j.1472-765X.1991.tb00608.x

[B11] Ash C. Priest F. Collins M. D. (1993). Molecular identification of rRNA group 3 bacilli (Ash, Farrow, Wallbanks and Collins) using a PCR probe test. Antonie Van Leeuwenhoek. 64, 253–260. doi: 10.1007/BF00873085 8085788

[B12] Ayaz M. Ali Q. Zhao W. Chi Y. K. Ali F. Rashid K. A.. (2024). Exploring plant growth promoting traits and biocontrol potential of new isolated *Bacillus subtilis* BS-2301 strain in suppressing. Front. Plant Sci. 15, 1444328. doi: 10.3389/fpls.2024.1444328 39239197 PMC11374654

[B13] Backer R. Mandolino G. Wilkins O. ElSohly M. A. Smith D. L. (2020). Cannabis genomics, breeding and production. Front. Plant Sci. 11, 591445. doi: 10.3389/fpls.2020.591445 33193546 PMC7661384

[B14] Backer R. Schwinghamer T. Rosenbaum P. McCarty V. Eichhorn Bilodeau S. Lyu D.. (2019). Closing the yield gap for cannabis: a meta-analysis of factors determining cannabis yield. Front. Plant Sci. 10, 495. doi: 10.3389/fpls.2019.00495 31068957 PMC6491815

[B15] Baedke J. Fábregas-Tejeda A. Nieves Delgado A. (2020). The holobiont concept before Margulis. J. Exp. Zoology Part B: Mol. Dev. Evol. 334, 149–155. doi: 10.1002/jez.b.v334.3 32039567

[B16] Balthazar C. Novinscak A. Cantin G. Joly D. L. Filion M. (2022). Biocontrol activity of *Bacillus* spp. and *Pseudomonas* spp. against *Botrytis cinerea* and other cannabis fungal pathogens. Phytopathology® 112, 549–560. doi: 10.1094/PHYTO-03-21-0128-R 34293909

[B17] Bertoni G. (2011). Indolebutyric acid–derived Auxin and plant development. Plant Cell 23, 845. doi: 10.1105/tpc.111.230312

[B18] Bhat M. A. Mishra A. K. Jan S. Bhat M. A. Kamal M. A. Rahman S.. (2023). Plant growth promoting rhizobacteria in plant health: a perspective study of the underground interaction. Plants 12, 629. doi: 10.3390/plants12030629 36771713 PMC9919780

[B19] Blanco Crivelli X. Cundon C. Bonino M. P. Sanin M. S. Bentancor A. (2024). The complex and changing cenus *Bacillus*: A diverse bacterial powerhouse for many applications. Bacteria 3, 256–270. doi: 10.3390/bacteria3030017

[B20] Boopathi N. M. Boopathi N. M. (2020). “Marker-assisted selection (MAS),” in Genetic mapping and marker assisted selection: Basics, practice and benefits, 343–388.

[B21] Bruni N. Della Pepa C. Oliaro-Bosso S. Pessione E. Gastaldi D. Dosio F. (2018). Cannabinoid delivery systems for pain and inflammation treatment. Molecules 23, 2478. doi: 10.3390/molecules23102478 30262735 PMC6222489

[B22] Chandra H. Kumari P. Bisht R. Prasad R. Yadav S. (2020). Plant growth promoting *Pseudomonas aeruginosa* from *Valeriana wallichii* displays antagonistic potential against three phytopathogenic fungi. Mol. Biol. Rep. 47, 6015–6026. doi: 10.1007/s11033-020-05676-0 32734439

[B23] Chen B. Han H. Hou J. Bao F. Tan H. Lou X.. (2022). Control of maize sheath blight and elicit induced systemic resistance using *Paenibacillus* polymyxa strain SF05. Microorganisms 10, 1318. doi: 10.3390/microorganisms10071318 35889037 PMC9322256

[B24] Chouhan U. Gamad U. Choudhari J. K. (2023). Metagenomic analysis of soybean endosphere microbiome to reveal signatures of microbes for health and disease. J. Genet. Eng. Biotechnol. 21, 84. doi: 10.1186/s43141-023-00535-4 37584775 PMC10429481

[B25] Comeau D. Balthazar C. Novinscak A. Bouhamdani N. Joly D. L. Filion M. (2021). Interactions between *Bacillus* spp., *Pseudomonas* spp. and *Cannabis sativa* promote plant growth. Front. Microbiol. 12, 715758. doi: 10.3389/fmicb.2021.715758 34616381 PMC8488376

[B26] Compant S. Cambon M. C. Vacher C. Mitter B. Samad A. Sessitsch A. (2021). The plant endosphere world–bacterial life within plants. Environ. Microbiol. 23, 1812–1829. doi: 10.1111/1462-2920.15240 32955144

[B27] Corredor-Perilla I. C. Andrade J. L. C. Olejar K. J. Park S. H. (2023). Beneficial properties of soil bacteria from Cannabis sativa L.: Seed germination, phosphorus solubilization and mycelial growth inhibition of Fusarium sp. Rhizosphere 27, 100780.

[B28] Cristino L. Bisogno T. Di Marzo V. (2020). Cannabinoids and the expanded endocannabinoid system in neurological disorders. Nat. Rev. Neurol. 16, 9–29. doi: 10.1038/s41582-019-0284-z 31831863

[B29] Cui W. Zhang J. Wang W. Wu X. Luo X. Zou Y.. (2024). Screening native *Bacillus* strains as potential biological control agents against ginger bacterial wilt and for promoting plant growth. Biol. Control 192, 105510. doi: 10.1016/j.biocontrol.2024.105510

[B30] Dasgupta D. Panda A. K. Mishra R. Mahanty A. De Mandal S. Bisht S. S. (2021). “ *Nif* genes: tools for sustainable agriculture,” in Recent Advancement in Microbial Biotechnology (Academic Press), 413–434.

[B31] De Andrade L. A. Santos C. H. B. Frezarin E. T. Sales L. R. Rigobelo E. C. (2023). Plant growth-promoting rhizobacteria for sustainable agricultural production. Microorganisms 11, 1088. doi: 10.3390/microorganisms11041088 37110511 PMC10146397

[B32] De Araújo F. de Araújo A. Figueiredo M. (2011). “Role of plant growth-promoting bacteria in sustainable agriculture,” in Sustainable agriculture: Technology, Planning and Management edit by Augusto Salazar and Ismael Rios (Nova Science Publishers, New York).

[B33] De Ron A. M. Álvarez-García S. Casquero P. A. Carro-Huelga G. Gutiérrez S. Lorenzana A. S.. (2024). “Clinical application and future consideration and potential of cannabis,” in Cannabis and Derivatives (Academic Press), 335–355.

[B34] Dhiman A. Mittal G. Tushir S. (2024). Clinical application and future consideration and potential of cannabis. Cannabis and Derivatives. Academic Press, 335–355

[B35] Di Y. N. Kui L. Singh P. Liu L. F. Xie L. Y. He L. L.. (2023). Identification and characterization of *Bacillus subtilis* B9: A diazotrophic plant growth-promoting endophytic bacterium isolated from sugarcane root. J. Plant Growth Regul. 42, 1720–1737. doi: 10.1007/s00344-022-10653-x

[B36] Dong C. J. Wang L. L. Li Q. Shang Q. M. (2019). Bacterial communities in the rhizosphere, phyllosphere and endosphere of tomato plants. PLoS One 14, e0223847. doi: 10.1371/journal.pone.0223847 31703074 PMC6839845

[B37] Dumigan C. R. Deyholos M. K. (2022). Cannabis seedlings inherit seed-borne bioactive and anti-fungal endophytic *Bacilli* . Plants 11, 2127. doi: 10.3390/plants11162127 36015430 PMC9415172

[B38] El-Saadony M. T. Saad A. M. Soliman S. M. Salem H. M. Ahmed A. I. Mahmood M.. (2022). Plant growth-promoting microorganisms as biocontrol agents of plant diseases: Mechanisms, challenges and future perspectives. Front. Plant Sci. 13, 923880. doi: 10.3389/fpls.2022.923880 36275556 PMC9583655

[B39] Etesami H. Adl S. M. (2020). Can interaction between silicon and non–rhizobial bacteria benefit in improving nodulation and nitrogen fixation in salinity–stressed legumes? A review. Rhizosphere, 100229. doi: 10.1016/j.rhisph.2020.100229

[B40] Etesami H. Jeong B. R. Glick B. R. (2023). Potential use of *Bacillus* spp. as an effective biostimulant against abiotic stresses in crops—A review. Curr. Res. Biotechnol. 5, 100128. doi: 10.1016/j.crbiot.2023.100128

[B41] Fahde S. Boughribil S. Sijilmassi B. Amri A. (2023). Rhizobia: a promising source of plant growth-promoting molecules and their non-legume interactions: examining applications and mechanisms. Agriculture 13, 1279. doi: 10.3390/agriculture13071279

[B42] Fakruddin M. Bin Mannan K. S. Mazumdar R. M. Chowdhury A. Hossain N. (2013). Identification and characterization of microorganisms:DNA-fingerprinting methods. Songklanakarin J. Sci. Technol. 35.

[B43] Fan P. Chen D. He Y. Zhou Q. Tian Y. Gao L. (2016). Alleviating salt stress in tomato seedlings using *Arthrobacter* and *Bacillus megaterium* isolated from the rhizosphere of wild plants grown on saline–alkaline lands. Int. J. phytoremediation 18, 1113–1121. doi: 10.1080/15226514.2016.1183583 27196364

[B44] Fan D. Schwinghamer T. Liu S. Xia O. Ge C. Chen Q.. (2023). Characterization of endophytic bacteriome diversity and associated beneficial bacteria inhabiting a macrophyte *Eichhornia crassipes* . Front. Plant Sci. 14, 1176648. doi: 10.3389/fpls.2023.1176648 37404529 PMC10316030

[B45] Fan D. Schwinghamer T. Smith D. L. (2018). Isolation and diversity of culturable rhizobacteria associated with economically important crops and uncultivated plants in Québec, Canada. Systematic Appl. Microbiol. 41, 629–640. doi: 10.1016/j.syapm.2018.06.004 30055880

[B46] Fernández-Llamosas H. Ibero J. Thijs S. Imperato V. Vangronsveld J. Díaz E.. (2020). Enhancing the rice seedlings growth promotion abilities of Azoarcus sp. CIB by heterologous expression of ACC deaminase to improve performance of plants exposed to cadmium stress. Microorganisms 8, 1453. doi: 10.3390/microorganisms8091453 32971998 PMC7564240

[B47] Firrincieli A. Khorasani M. Frank A. C. Doty S. L. (2020). Influences of climate on phyllosphere endophytic bacterial communities of wild poplar. Front. Plant Sci. 11, 203. doi: 10.3389/fpls.2020.00203 32184800 PMC7058686

[B48] Gómez-De la Cruz I. Chávez-Ramírez B. Avendaño-Arrazate C. H. Morales-García Y. E. Muñoz-Rojas J. Estrada-de Los Santos P. (2023). Optimization of *Paenibacillus* sp. NMA1017 Application as a biocontrol agent for *Phytophthora tropicalis* and *Moniliophthora roreri* in cacao-growing fields in Chiapas, Mexico. Plants 12, 2336. doi: 10.3390/plants12122336 37375961 PMC10301746

[B49] Govindasamy V. Senthilkumar M. Magheshwaran V. Kumar U. Bose P. Sharma V.. (2011). *Bacillus* and *Paenibacillus* spp.: potential PGPR for sustainable agriculture. Plant Growth Health promoting bacteria, 333–364.

[B50] Grady E. N. MacDonald J. Liu L. Richman A. Yuan Z. C. (2016). Current knowledge and perspectives of *Paenibacillus*: a review. Microbial Cell factories 15, 1–18. doi: 10.1186/s12934-016-0603-7 27905924 PMC5134293

[B51] Hassan M. K. McInroy J. A. Kloepper J. W. (2019). The interactions of rhizodeposits with plant growth-promoting rhizobacteria in the rhizosphere: a review. Agriculture 9, 142. doi: 10.3390/agriculture9070142

[B52] Hyder S. Gondal A. S. Riaz N. Rashid M. Qaiser Z. de los Santos-Villalobos S.. (2024). “Plant growth promoting rhizobacteria (PGPR): A green approach to manage soil-borne fungal pathogens and plant growth promotion,” in Microbial Technology for Agro-Ecosystems (Academic Press), 153–176.

[B53] Iturralde E. T. Covelli J. M. Alvarez F. Pérez-Giménez J. Arrese-Igor C. Lodeiro A. R. (2019). Soybean-nodulating strains with low intrinsic competitiveness for nodulation, good symbiotic performance, and stress-tolerance isolated from soybean-cropped soils in Argentina. Front. Microbiol. 10, 1061. doi: 10.3389/fmicb.2019.01061 31139173 PMC6527597

[B54] Jain T. Gehlot P. Yadav J. Chittora D. (2023). Molecular basis of biotic and abiotic stress management attributes of plant growth promoting rhizobacteria. J. Postharvest Technol. 11, 29–55.

[B55] Jain S. Varma A. Choudhary D. K. (2021). Perspectives on nitrogen-fixing *Bacillus* species. Soil Nitrogen Ecol., 359–369.

[B56] Jalali S. Salami S. A. Sharifi M. Sohrabi S. (2019). Signaling compounds elicit expression of key genes in cannabinoid pathway and related metabolites in cannabis. Ind. Crops Products 133, 105–110. doi: 10.1016/j.indcrop.2019.03.004

[B57] Janczarek A. Gałązka A. (2024). Genetic methods of identification, classification, and differentiation of bacteria. Microbial Genet., 125–137.

[B58] Jiang C. H. Liao M. J. Wang H. K. Zheng M. Z. Xu J. J. Guo J. H. (2018). *Bacillus velezensis*, a potential and efficient biocontrol agent in control of pepper gray mold caused by *Botrytis cinerea* . Biol. Control 126, 147–157. doi: 10.1016/j.biocontrol.2018.07.017

[B59] Kamle M. Borah R. Bora H. Jaiswal A. K. Singh R. K. Kumar P. (2020). “Systemic acquired resistance (SAR) and induced systemic resistance (ISR): role and mechanism of action against phytopathogens,” in Fungal biotechnology and bioengineering (Springer, Cham), 457–470.

[B60] Kang W. S. Chen L. J. Wang Y. Y. Zhu X. F. Liu X. Y. Fan H. Y.. (2020). *Bacillus simplex* treatment promotes soybean defense against soybean cyst nematodes: A metabolomics study using GC-MS. PLoS One 15, e0237194. doi: 10.1371/journal.pone.0237194 32760135 PMC7410315

[B61] Kaymak H. Ürüşan A. Tiraşci S. Kaşka M. (2023). Role of N2-fixing plant growth-promoting rhizobacteria in some selected vegetables. Türk Tarım-Gıda Bilim ve Teknoloji dergisi 11. doi: 10.24925/turjaf.v11i6.1183-1194.6033

[B62] Khan A. L. (2023). The phytomicrobiome: solving plant stress tolerance under climate change. Front. Plant Sci. 14, 1219366. doi: 10.3389/fpls.2023.1219366 37746004 PMC10513501

[B63] Khan M. S. Gao J. Chen X. Zhang M. Yang F. Du Y.. (2020). Isolation and characterization of plant growth-promoting endophytic bacteria *Paenibacillus polymyxa* SK1 from *Lilium lancifolium* . BioMed. Res. Int. 2020, 8650957. doi: 10.1155/2020/8650957 32190683 PMC7064867

[B64] Khodashenas Rudsari M. Zouhar M. Manasova M. Li T. (2024). Biocontrol potential of cell-free supernatant of *Paenibacillus chitinolyticus* against *Plasmodiophora brassicae* in two important *Brassica* species. Eur. J. Plant Pathol., 1–12. doi: 10.21203/rs.3.rs-3769218/v1

[B65] Kour D. Yadav A. N. (2022). Bacterial mitigation of drought stress in plants: Current perspectives and future challenges. Curr. Microbiol. 79, 248. doi: 10.1007/s00284-022-02939-w 35834053

[B66] Kousar B. Bano A. Khan N. (2020). PGPR modulation of secondary metabolites in tomato infested with *Spodoptera litura* . Agronomy 10, 778. doi: 10.3390/agronomy10060778

[B67] Kumar S. Anjali Arutselvan R. Masurkar P. Singh U. B. Tripathi R.. (2024). “ *Bacillus subtilis*-mediated induction of disease resistance and promotion of plant growth of vegetable crops,” in Applications of *Bacillus* and *Bacillus* Derived Genera in Agriculture, Biotechnology and Beyond (Springer Nature Singapore, Singapore), 165–211.

[B68] Kumawat K. C. Sharma B. Nagpal S. Kumar A. Tiwari S. Nair R. M. (2023). Plant growth-promoting rhizobacteria: Salt stress alleviators to improve crop productivity for sustainable agriculture development. Front. Plant Sci. 13, 1101862. doi: 10.3389/fpls.2022.1101862 36714780 PMC9878403

[B69] Kwon Y. S. Lee D. Y. Rakwal R. Baek S. B. Lee J. H. Kwak Y. S.. (2016). Proteomic analyses of the interaction between the plant-growth promoting rhizobacterium *Paenibacillus polymyxa* E681 and *Arabidopsis thaliana* . Proteomics 16, 122–135. doi: 10.1002/pmic.201500196 26460066

[B70] Lee Y. Balaraju K. Kim S. Y. Jeon Y. (2022). Occurrence of phenotypic variation in *Paenibacillus polymyxa* E681 associated with sporulation and carbohydrate metabolism. Biotechnol. Rep. 34, e00719. doi: 10.1016/j.btre.2022.e00719 PMC9171445 35686012

[B71] Legein M. Smets W. Vandenheuvel D. Eilers T. Muyshondt B. Prinsen E.. (2020). Modes of action of microbial biocontrol in the phyllosphere. Front. Microbiol., 1619. doi: 10.3389/fmicb.2020.01619 32760378 PMC7372246

[B72] Li H. P. Gan Y. N. Yue L. J. Han Q. Q. Chen J. Liu Q. M.. (2022). Newly isolated Paenibacillus monticola sp. nov., a novel plant growth-promoting rhizobacteria strain from high-altitude spruce forests in the Qilian Mountains, China. Front. Microbiol. 13, 833313. doi: 10.3389/fmicb.2022.833313 35250949 PMC8895201

[B73] Li Y. Li Y. Zhang H. Wang M. Chen S. (2019). Diazotrophic *Paenibacillus beijingensis* BJ-18 provides nitrogen for plant and promotes plant growth, nitrogen uptake and metabolism. Front. Microbiol. 10, 1119. doi: 10.3389/fmicb.2019.01119 31191471 PMC6548809

[B74] Li Y. Wang M. Chen S. (2021). Application of N_2_-fixing *Paenibacillus triticisoli* BJ-18 changes the compositions and functions of the bacterial, diazotrophic, and fungal microbiomes in the rhizosphere and root/shoot endosphere of wheat under field conditions. Biol. Fertility Soils 57, 347–362. doi: 10.1007/s00374-020-01528-y

[B75] Li Q. Zhang H. Zhang L. Chen S. (2021). Functional analysis of multiple *nifB* genes of *Paenibacillus* strains in synthesis of Mo-, Fe-and V-nitrogenases. Microbial Cell Factories 20, 1–14. doi: 10.1186/s12934-021-01629-9 34281551 PMC8287671

[B76] Liu W. Sikora E. Park S. W. (2020). Plant growth-promoting rhizobacterium, *Paenibacillus polymyxa* CR1, upregulates dehydration-responsive genes, RD29A and RD29B, during priming drought tolerance in *Arabidopsis* . Plant Physiol. Biochem. 156, 146–154. doi: 10.1016/j.plaphy.2020.08.049 32947123

[B77] Logan N. A. Halket G. (2011). “Developments in the taxonomy of aerobic, endospore-forming bacteria,” in Endospore-forming Soil Bacteria (Springer Berlin Heidelberg, Berlin, Heidelberg), 1–29.

[B78] Louws F. J. Fulbright D. W. Stephens C. T. De Bruijn F. J. (1995). Differentiation of genomic structure by rep-PCR finger-printing to rapidly classify Xanthomonas campestris pv. vesicatoria. Phytopathology 5 (5), 528–536.

[B79] Lyu D. Backer R. Berrué F. Martinez-Farina C. Hui J. P. Smith D. L. (2023). Plant growth-promoting rhizobacteria (PGPR) with microbial growth broth improve biomass and secondary metabolite accumulation of *Cannabis sativa* L. J. Agric. Food Chem. 71, 7268–7277. doi: 10.1021/acs.jafc.2c06961 37130078 PMC10197126

[B80] Lyu D. Backer R. Smith D. L. (2022). Three plant growth-promoting rhizobacteria alter morphological development, physiology, and flower yield of *Cannabis sativa* L. Ind. Crops Products 178, 114583. doi: 10.1016/j.indcrop.2022.114583

[B81] Lyu D. Zajonc J. Pagé A. Tanney C. A. Shah A. Monjezi N.. (2021). Plant holobiont theory: the phytomicrobiome plays a central role in evolution and success. Microorganisms 9, 675. doi: 10.3390/microorganisms9040675 33805166 PMC8064057

[B82] Ma X. Xu Z. Lang D. Zhou L. Zhang W. Zhang X. (2022). Comprehensive physiological, transcriptomic, and metabolomic analyses reveal the synergistic mechanism of *Bacillus pumilus* G5 combined with silicon alleviate oxidative stress in drought-stressed *Glycyrrhiza uralensis* Fisch. Front. Plant Sci. 13, 1033915. doi: 10.3389/fpls.2022.1033915 36570944 PMC9773211

[B83] Mahapatra S. Chakraborty S. Samanta M. Das S. Islam T. (2022). “Current understanding and future directions of biocontrol of plant diseases by Bacillus spp., with special reference to induced systemic resistance,” in *Bacilli* in Agrobiotechnology: Plant Stress Tolerance, Bioremediation, and Bioprospecting (Springer International Publishing, Cham), 127–150.

[B84] Mansouri H. Asrar Z. Amarowicz R. (2011). The response of terpenoids to exogenous gibberellic acid in *Cannabis sativa* L. at vegetative stage. Acta Physiologiae Plantarum 33, 1085–1091. doi: 10.1007/s11738-010-0636-1

[B85] Matys J. Kensy J. Gedrange T. Zawiślak I. Grzech-Leśniak K. Dobrzyński M. (2024). A molecular approach for detecting bacteria and fungi in healthcare environment aerosols: A systematic review. Int. J. Mol. Sci. 25, 4154. doi: 10.3390/ijms25084154 38673740 PMC11050369

[B86] Mehta M. P. Butterfield D. A. Baross J. A. (2003). Phylogenetic diversity of nitrogenase (*nifH*) genes in deep-sea and hydrothermal vent environments of the Juan de Fuca Ridge. Appl. Environ. Microbiol. 69, 960–970. doi: 10.1128/AEM.69.2.960-970.2003 12571018 PMC143675

[B87] Miljaković D. Marinković J. Balešević-Tubić S. (2020). The significance of Bacillus spp. in disease suppression and growth promotion of field and vegetable crops. Microorganisms 8, 1037. doi: 10.3390/microorganisms8071037 32668676 PMC7409232

[B88] Mitra B. Chowdhury A. R. Dey P. Hazra K. K. Sinha A. K. Hossain A.. (2021). “Use of agrochemicals in agriculture: alarming issues and solutions,” in Input use efficiency for food and environmental security, 85–122.

[B89] Moosa A. Zulfiqar F. Alalawy A. I. Almowallad S. Al-Massabi R. F. (2024). Transcriptional and biochemical profiling of *Bacillus* strains regulating the growth of tomato via altering morpho-physiological and hormonal traits. Scientia Hortic. 328, 112881. doi: 10.1016/j.scienta.2024.112881

[B90] Morcillo R. J. Manzanera M. (2021). The effects of plant-associated bacterial exopolysaccharides on plant abiotic stress tolerance. Metabolites 11, 337. doi: 10.3390/metabo11060337 34074032 PMC8225083

[B91] Moreno-Serrano D. Gaines T. A. Dayan F. E. (2024). Current status of auxin-mimic herbicides. Outlooks Pest Manage. 35, 105–112. doi: 10.1564/v35_jun_04

[B92] Mun B. G. Hussain A. Park Y. G. Kang S. M. Lee I. J. Yun B. W. (2024). The PGPR *Bacillus aryabhatta*i promotes soybean growth via nutrient and chlorophyll maintenance and the production of butanoic acid. Front. Plant Sci. 15, 1341993. doi: 10.3389/fpls.2024.1341993 38439982 PMC10909845

[B93] Naamala J. Msimbira L. A. Antar M. Subramanian S. Smith D. L. (2022). Cell-free supernatant obtained from a salt tolerant *Bacillus amyloliquefaciens* strain enhances germination and radicle length under NaCl stressed and optimal conditions. Front. Sustain. Food Syst. 6, 788939. doi: 10.3389/fsufs.2022.788939

[B94] Nazari M. Smith D. L. (2020). A PGPR-produced bacteriocin for sustainable agriculture: a review of thuricin 17 characteristics and applications. Front. Plant Sci. 11, 916. doi: 10.3389/fpls.2020.00916 32733506 PMC7358586

[B95] Nie P. Li X. Wang S. Guo J. Zhao H. Niu D. (2017). Induced systemic resistance against *Botrytis cinerea* by *Bacillus cereus* AR156 through a JA/ET-and NPR1-dependent signaling pathway and activates PAMP-triggered immunity in *Arabidopsis* . Front. Plant Sci. 8, 238. doi: 10.3389/fpls.2017.00238 28293243 PMC5329000

[B96] Nifakos K. Tsalgatidou P. C. Thomloudi E. E. Skagia A. Kotopoulis D. Baira E.. (2021). Genomic analysis and secondary metabolites production of the endophytic *Bacillus velezensis* Bvel1: A biocontrol agent against *Botrytis cinerea* causing bunch rot in post-harvest table grapes. Plants 10, 1716. doi: 10.3390/plants10081716 34451760 PMC8400388

[B97] Nuraini Y. Ustiatik R. Chasanah U. Ekanayake W. (2024). “Improving soil fertility and soybean production by growth-promoting bacteria in drylands,” in Applied Ecology & Environmental Research, vol. 22. .

[B98] Odoh C. K. Sam K. Zabbey N. Eze C. N. Nwankwegu A. S. Laku C.. (2020). “Microbial consortium as biofertilizers for crops growing under the extreme habitats,” in Plant Microbiomes for Sustainable Agriculture (Springer, Cham), 381–424.

[B99] Olishevska S. Nickzad A. Déziel E. (2019). *Bacillus* and *Paenibacillus* secreted polyketides and peptides involved in controlling human and plant pathogens. Appl. Microbiol. Biotechnol. 103, 1189–1215. doi: 10.1007/s00253-018-9541-0 30603850

[B100] Orozco-Mosqueda M. D. C. Flores A. Rojas-Sánchez B. Urtis-Flores C. A. Morales-Cedeño L. R. Valencia-Marin M. F.. (2021). Plant growth-promoting bacteria as bioinoculants: attributes and challenges for sustainable crop improvement. Agronomy 11, 1167. doi: 10.3390/agronomy11061167

[B101] Pandey V. V. Bhattacharya A. Pandey A. (2023). “Plant growth-promoting microbiomes: History and their role in agricultural crop improvement,” in Plant-Microbe Interaction-Recent Advances in Molecular and Biochemical Approaches, 1–44.

[B102] Parte A. C. (2014). LPSN—list of prokaryotic names with standing in nomenclature. Nucleic Acids Res. 42, D613–D616. doi: 10.1093/nar/gkt1111 24243842 PMC3965054

[B103] Pastor-Bueis R. Jiménez-Gómez A. Barquero M. Mateos P. F. González-Andrés F. (2021). Yield response of common bean to co-inoculation with Rhizobium and Pseudomonas endophytes and microscopic evidence of different colonised spaces inside the nodule. European Journal of Agronomy. 122, 126187

[B104] Patel S. Gupta R. S. (2020). A phylogenomic and comparative genomic framework for resolving the polyphyly of the genus Bacillus: Proposal for six new genera of Bacillus species, *Peribacillus* gen. nov., *Cytobacillus* gen. nov., *Mesobacillus* gen. nov., *Neobacillus* gen. nov., *Metabacillus* gen. nov. and *Alkalihalobacillus* gen. nov. Int. J. systematic evolutionary Microbiol. 70, 406–438. doi: 10.1099/ijsem.0.003775 31617837

[B105] Pattnaik S. Mohapatra B. Kumar U. Pattnaik M. Samantaray D. (2019). “Microbe-mediated plant growth promotion: a mechanistic overview on cultivable plant growth-promoting members,” in Biofertilizers for sustainable agriculture and environment, 435–463.

[B106] Prabhukarthikeyan S. R. Keerthana U. Baite M. S. Panneerselvam P. Mitra D. Kumar R. N.. (2022). “ *Bacillus* rhizobacteria: A versatile biostimulant for sustainable agriculture,” in New and future developments in microbial biotechnology and bioengineering (Elsevier), 33–44.

[B107] Priest F. G. Goodfellow M. Todd C. (1988). A numerical classification of the genus *Bacillus* . Microbiology 134, 1847–1882. doi: 10.1099/00221287-134-7-1847 3246588

[B108] Prudent M. Salon C. Souleimanov A. Emery R. N. Smith D. L. (2015). Soybean is less impacted by water stress using *Bradyrhizobium japonicum* and thuricin-17 from *Bacillus thuringiensis* . Agron. Sustain. Dev. 35, 749–757. doi: 10.1007/s13593-014-0256-z

[B109] Qin S. Li J. Chen H. H. Zhao G. Z. Zhu W. Y. Jiang C. L.. (2009). Isolation, diversity, and antimicrobial activity of rare actinobacteria from medicinal plants of tropical rain forests in Xishuangbanna, China. Appl. Environ. Microbiol. 75, 6176–6186. doi: 10.1128/AEM.01034-09 19648362 PMC2753051

[B110] Rademaker J. L. W. Louws F. J. Versalovic J. A. M. E. S. de Bruijn F. J. Kowalchuk G. A. (2004). Characterization of the diversity of ecologically important microbes by rep-PCR genomic fingerprinting. Mol. microbial Ecol. manual 1, 611–643.

[B111] Rampitsch C. Bykova N. V. (2012). Proteomics and plant disease: advances in combating a major threat to the global food supply. Proteomics 12, 673–690. doi: 10.1002/pmic.201100359 22246663

[B112] Rana K. L. Kour D. Kaur T. Negi R. Devi R. Yadav N.. (2023). Endophytic nitrogen-fixing bacteria: untapped treasurer for agricultural sustainability. J. Appl. Biol. Biotechnol. 11, 75–93. doi: 10.7324/JABB.2023.110207

[B113] Renganathan P. (2020). “Chapter-9 bacterial bioprotectants for the management of sesame leaf spot in rice,” in Current Research and Innovations in Plant Pathology, 163.

[B114] Ribeiro I. D. A. Paes J. A. Wendisch V. F. Ferreira H. B. Passaglia L. M. P. (2024). Proteome profiling of *Paenibacillus sonchi* genomovar *Riograndensis* SBR5T under conventional and alternative nitrogen fixation. J. Proteomics 294, 105061. doi: 10.1016/j.jprot.2023.105061 38154550

[B115] Rosli M. A. Azam Z. M. Othman N. Z. Sarmidi M. R. (2020). *Paenibacillus polymyxa* role involved in phosphate solubilization and growth promotion of *Zea mays* under abiotic stress condition. Proc. Natl. Acad. Sciences India Section B: Biol. Sci. 90, 63–71.

[B116] Saeed Q. Xiukang W. Haider F. U. Kučerik J. Mumtaz M. Z. Holatko J.. (2021). Rhizosphere bacteria in plant growth promotion, biocontrol, and bioremediation of contaminated sites: a comprehensive review of effects and mechanisms. Int. J. Mol. Sci. 22, 10529. doi: 10.3390/ijms221910529 34638870 PMC8509026

[B117] Salvatierra-Martinez R. Arancibia W. Araya M. Aguilera S. Olalde V. Bravo J.. (2018). Colonization ability as an indicator of enhanced biocontrol capacity—An example using two *Bacillus amyloliquefaciens* strains and *Botrytis cinerea* infection of tomatoes. J. Phytopathol. 166, 601–612. doi: 10.1111/jph.12718

[B118] Schulz-Bohm K. Martín-Sánchez L. Garbeva P. (2017). Microbial volatiles: small molecules with an important role in intra-and inter-kingdom interactions. Front. Microbiol. 8, 2484. doi: 10.3389/fmicb.2017.02484 29312193 PMC5733050

[B119] Scott M. Rani M. Samsatly J. Charron J. B. Jabaji S. (2018). Endophytes of industrial hemp (*Cannabis sativa* L.) cultivars: identification of culturable bacteria and fungi in leaves, petioles, and seeds. Can. J. Microbiol. 64, 664–680. doi: 10.1139/cjm-2018-0108 29911410

[B120] Shi Q. Zhang J. Fu Q. Hao G. Liang C. Duan F.. (2024). Biocontrol efficacy and induced resistance of *Paenibacillus polymyxa* J2–4 against *Meloidogyne incognita* infection in cucumber. Phytopathology® 114, 538–548. doi: 10.1094/PHYTO-03-23-0091-R 37698495

[B121] Soares M. B. Almada C. N. Pereira E. P. Ferreira B. M. Balthazar C. F. Khorshidian N.. (2023). Sporeforming probiotic bacteria: Characteristics, health benefits, and technological aspects for their applications in foods and beverages. Trends in Food Science & Technology 138, 453–469.

[B122] Shida O. Takagi H. Kadowaki K. Nakamura L. K. Komagata K. (1997). Transfer of *Bacillus alginolyticus*, *Bacillus chondroitinus*, *Bacillus curdlanolyticus*, *Bacillus glucanolyticus*, *Bacillus kobensis*, and *Bacillus thiaminolyticus* to the genus *Paenibacillus* and emended description of the genus *Paenibacillus* . Int. J. Systematic Bacteriol 47, 289–298. doi: 10.1099/00207713-47-2-289 9103612

[B123] Soy Canada (2023). Growing areas. Available online at: https://soycanada.ca/iindustry/growing-areas/ (Accessed July 5, 2023).

[B124] Subramanian S. Mitkus E. Souleimanov A. Smith D. L. (2023). Lipo-chitooligosaccharide and thuricin 17 act as plant growth promoters and alleviate drought stress in *Arabidopsis thaliana* . Front. Microbiol. 14, 1184158. doi: 10.3389/fmicb.2023.1184158 37601342 PMC10436337

[B125] Subramanian S. Ricci E. Souleimanov A. Smith D. L. (2016). A proteomic approach to lipo-chitooligosaccharide and thuricin 17 effects on soybean germination and salt stress. PloS One 11, e0160660. doi: 10.1371/journal.pone.0160660 27560934 PMC4999219

[B126] Subramanian S. Smith D. L. (2015). Bacteriocins from the rhizosphere microbiome–from an agriculture perspective. Front. Plant Sci. 6, 909. doi: 10.3389/fpls.2015.00909 26579159 PMC4626563

[B127] Suleiman M. K. Quoreshi A. M. Bhat N. R. Manuvel A. J. Sivadasan M. T. (2019). Divulging diazotrophic bacterial community structure in Kuwait desert ecosystems and their N2-fixation potential. PloS One 14, e0220679. doi: 10.1371/journal.pone.0220679 31877136 PMC6932743

[B128] Sun L. Wang W. Zhang X. Gao Z. Cai S. Wang S.. (2023). *Bacillus velezensis* BVE7 as a promising agent for biocontrol of soybean root rot caused by *Fusarium oxysporum* . Front. Microbiol. 14, 1275986. doi: 10.3389/fmicb.2023.1275986 37928669 PMC10623355

[B129] Sun H. Zhang J. Liu W. E W. Wang X. Li H.. (2022). Identification and combinatorial engineering of indole-3-acetic acid synthetic pathways in *Paenibacillus polymyxa* . Biotechnol. Biofuels Bioproducts 15, 81. doi: 10.1186/s13068-022-02181-3 PMC9367139 35953838

[B130] Taghinasab M. Jabaji S. (2020). Cannabis microbiome and the role of endophytes in modulating the production of secondary metabolites: an overview. Microorganisms 8, 355. doi: 10.3390/microorganisms8030355 32131457 PMC7143057

[B131] Taheri E. Tarighi S. Taheri P. (2022). Characterization of root endophytic *Paenibacillus polymyxa* isolates with biocontrol activity against *Xanthomonas translucens* and *Fusarium graminearum* . Biol. Control 174, 105031. doi: 10.1016/j.biocontrol.2022.105031

[B132] Tanaka K. Heil M. (2021). Damage-associated molecular patterns (DAMPs) in plant innate immunity: applying the danger model and evolutionary perspectives. Annu. Rev. Phytopathol. 59, 53–75. doi: 10.1146/annurev-phyto-082718-100146 33900789

[B133] Tanney C. A. Lyu D. Schwinghamer T. Geitmann A. Ruan E. D. Smith D. L. (2023). Sub-optimal nutrient regime coupled with *Bacillus* and Pseudomonas sp. inoculation influences trichome density and cannabinoid profiles in drug-type *Cannabis sativa* . Front. Plant Sci. 14, 1131346. doi: 10.3389/fpls.2023.1131346 37275248 PMC10236210

[B134] Tao H. Wang S. Li X. Li X. Cai J. Zhao L.. (2023). Biological control of potato common scab and growth promotion of potato by *Bacillus velezensis* Y6. Front. Microbiol. 14, 1295107. doi: 10.3389/fmicb.2023.1295107 38149275 PMC10750399

[B135] Toral L. Rodríguez M. Béjar V. Sampedro I. (2020). Crop protection against *Botrytis cinerea* by rhizhosphere biological control agent *Bacillus velezensis* XT1. Microorganisms 8, 992. doi: 10.3390/microorganisms8070992 32635146 PMC7409083

[B136] Tran D. M. Huynh T. U. Do T. O. Nguyen A. D. (2024). Isolation, plant growth-promoting properties, and whole-genome sequence of a novel *Paenibacillus* species. J. Basic Microbiol., e202400119. doi: 10.1002/jobm.202400119 38894514

[B137] Vasilchenko A. S. Valyshev A. V. (2019). Pore-forming bacteriocins: structural-functional relationships. Arch. Microbiol. 201, 147–154. doi: 10.1007/s00203-018-1610-3 30554292

[B138] Versalovic J. Koeuth T. Lupski R.. (1991). Distribution of repetitive DNA sequences in eubacteria and application to finerpriting of bacterial enomes. Nucleic Acids Res. 19 (24), 6823–6831. 10.1093/nar/19.24.6823 PMC329316 1762913

[B139] Vetterlein D. Carminati A. Kögel-Knabner I. Bienert G. P. Smalla K. Oburger E.. (2020). Rhizosphere spatiotemporal organization–a key to rhizosphere functions. Front. Agron. 2, 8. doi: 10.3389/fagro.2020.00008

[B140] Vimal S. R. Singh J. S. Prasad S. M. (2024). “Crop microbiome dynamics in stress management and green agriculture,” in Microbiome Drivers of Ecosystem Function (Academic Press), 341–366.

[B141] Vitorino L. C. da Silva E. J. Oliveira M. S. Silva I. D. O. Santos L. D. S. Mendonça M. A. C.. (2024). Effect of a *Bacillus velezensis* and *Lysinibacillus fusiformis*-based biofertilizer on phosphorus acquisition and grain yield of soybean. Front. Plant Sci. 15, 1433828. doi: 10.3389/fpls.2024.1433828 39246810 PMC11378753

[B142] Volkova E. Smolyaninova N. (2024). “Analysis of world trends in soybean production,” in BIO Web of Conferences, vol. 141. (EDP Sciences), 01026.

[B143] Wahab A. Muhammad M. Munir A. Abdi G. Zaman W. Ayaz A.. (2023). Role of arbuscular mycorrhizal fungi in regulating growth, enhancing productivity, and potentially influencing ecosystems under abiotic and biotic stresses. Plants 12, 3102. doi: 10.3390/plants12173102 37687353 PMC10489935

[B144] Wang S. Y. Herrera-Balandrano D. D. Wang Y. X. Shi X. C. Chen X. Jin Y.. (2022). Biocontrol Ability of the *Bacillus amyloliquefaciens* Group, *B. amyloliquefaciens*, *B. velezensis, B. nakamurai and B. siamensis*, for the Management of Fungal Postharvest Diseases: A Review. J. Agric. Food Chem. 10.1021/acs.jafc.2c01745 35604328

[B145] Wang J. Peng Y. Xie S. Yu X. Bian C. Wu H.. (2023). Biocontrol and molecular characterization of *Bacillus velezensis* D against tobacco bacterial wilt. Phytopathol. Res. 5, 50. doi: 10.1186/s42483-023-00204-x

[B146] Wang D. Poinsot V. Li W. Lu Y. Liu C. Li Y.. (2023). Genomic insights and functional analysis reveal plant growth promotion traits of *Paenibacillus mucilaginosus* G78. Genes 14, 392. doi: 10.3390/genes14020392 36833318 PMC9956331

[B147] Weselowski B. Nathoo N. Eastman A. W. MacDonald J. Yuan Z. C. (2016). Isolation, identification and characterization of *Paenibacillus polymyxa* CR1 with potentials for biopesticide, biofertilization, biomass degradation and biofuel production. BMC Microbiol. 16, 1–10. doi: 10.1186/s12866-016-0860-y 27756215 PMC5069919

[B148] Williams J. G. Kubelik A. R. Livak K. J. Rafalski J. A. Tingey S. V. (1990). DNA polymorphisms amplified by arbitrary primers are useful as genetic markers. Nucleic Acids Research 18 (22), 6531–6535. 1979162 10.1093/nar/18.22.6531 PMC332606

[B149] Winston M. E. Hampton-Marcell J. ZarraonaIndia I. Owens S. M. Moreau C. S. Gilbert J. A.. (2014). Understanding cultivar-specificity and soil determinants of the cannabis microbiome. PloS One 9, e99641. doi: 10.1371/journal.pone.0099641 24932479 PMC4059704

[B150] Xing P. Zhao Y. Guan D. Li L. Zhao B. Ma M.. (2022). Effects of *Bradyrhizobium* co-inoculated with *Bacillus* and *Paenibacillus* on the structure and functional genes of soybean Rhizobacteria community. Genes 13, 1922. doi: 10.3390/genes13111922 36360159 PMC9689485

[B151] Xu X. Kovács Á.T. (2024). How to identify and quantify the members of the *Bacillus* genus? Environ. Microbiol. 26, e16593 38383138 10.1111/1462-2920.16593

[B152] Yang F. Jiang H. Ma K. Hegazy A. Wang X. Liang S.. (2024). Genomic and phenotypic analyses reveal *Paenibacillus polymyxa* PJH16 is a potential biocontrol agent against cucumber *Fusarium* wilt. Front. Microbiol. 15, 1359263. doi: 10.3389/fmicb.2024.1359263 38591040 PMC11000672

[B153] Zeigler D. R. (2013). “The family *Paenibacillacea* ,” in Strain catalog and reference (*Bacillus* Genetic Stock Center, Columbus), 1–32.

[B154] Zeng Q. Ding X. Wang J. Han X. Iqbal H. M. Bilal M. (2022). Insight into soil nitrogen and phosphorus availability and agricultural sustainability by plant growth-promoting rhizobacteria. Environ. Sci. pollut. Res. 29, 45089–45106. doi: 10.1007/s11356-022-20399-4 35474421

[B155] Zhang C. Xie Y. He P. Shan L. (2024). Unlocking nature’s defense: plant pattern recognition receptors as guardians against pathogenic threats. Mol. Plant-Microbe Interact. 37, 73–83. doi: 10.1094/MPMI-10-23-0177-HH 38416059 PMC12643533

[B156] Zoundji M. C. C. Houngnandan P. Boko F. Toukourou F. (2020). Characterization of indigenous *Rhizobia* strains associated to soybean [*Glycine max* (L.) Merrill] in Benin. Int. J. Plant Soil Sci., 35–46. doi: 10.9734/ijpss/2020/v32i230244

